# A miR-375/YAP axis regulates neuroendocrine differentiation and tumorigenesis in lung carcinoid cells

**DOI:** 10.1038/s41598-021-89855-4

**Published:** 2021-05-17

**Authors:** Xiaojing Yang, Jina Nanayakkara, Duncan Claypool, Sadegh Saghafinia, Justin J. M. Wong, Minqi Xu, Xiantao Wang, Christopher J. B. Nicol, Iacovos P. Michael, Markus Hafner, Xiaolong Yang, Neil Renwick

**Affiliations:** 1 Laboratory of Translational RNA Biology, Department of Pathology and Molecular Medicine, Queen’s University, 88 Stuart St, Kingston, ON K7L 3N6 Canada; 2 Laboratory of Muscle Stem Cells and Gene Regulation, NIAMS, 50 South Drive, Bethesda, MD 20892 USA; 3 Swiss Institute for Experimental Cancer Research, School of Life Sciences, École Polytechnique Fédérale de Lausanne, 1015 Lausanne, Switzerland; 4 Department of Pathology and Molecular Medicine, Queen’s University, 88 Stuart St, Kingston, ON K7L 3N6 Canada; 5 Division of Cancer Biology and Genetics, Queen’s Cancer Research Institute, 10 Stuart St, Kingston, ON K7L 3N6 Canada; 6 Cancer Research Laboratory, Department of Pathology and Molecular Medicine, Queen’s University, 88 Stuart St, Kingston, ON K7L 3N6 Canada

**Keywords:** miRNAs, Neuroendocrine cancer

## Abstract

Lung carcinoids are variably aggressive and mechanistically understudied neuroendocrine neoplasms (NENs). Here, we identified and elucidated the function of a miR-375/yes-associated protein (YAP) axis in lung carcinoid (H727) cells. miR-375 and *YAP* are respectively high and low expressed in wild-type H727 cells. Following lentiviral CRISPR/Cas9-mediated miR-375 depletion, we identified distinct transcriptomic changes including dramatic YAP upregulation. We also observed a significant decrease in neuroendocrine differentiation and substantial reductions in cell proliferation, transformation, and tumor growth in cell culture and xenograft mouse disease models. Similarly, YAP overexpression resulted in distinct and partially overlapping transcriptomic changes, phenocopying the effects of miR-375 depletion in the same models as above. Transient YAP knockdown in miR-375-depleted cells reversed the effects of miR-375 on neuroendocrine differentiation and cell proliferation. Pathways analysis and confirmatory real-time PCR studies of shared dysregulated target genes indicate that this axis controls neuroendocrine related functions such as neural differentiation, exocytosis, and secretion. Taken together, we provide compelling evidence that a miR-375/YAP axis is a critical mediator of neuroendocrine differentiation and tumorigenesis in lung carcinoid cells.

## Introduction

Lung carcinoids are increasingly common and variably aggressive neuroendocrine neoplasms (NENs) that are incompletely understood at the molecular level^1–4^. Two pathological types, namely typical and atypical carcinoid (TC and AC), are currently recognized based on their mitotic activity and metastatic potential. Once metastatic, carcinoids are challenging to treat due to their high resistance to radiotherapy and chemotherapy^1,5^. In comparison with more aggressive lung NENs, namely small cell lung carcinoma (SCLC) and large cell neuroendocrine carcinoma (LCNEC), few studies focus on carcinoids and only limited therapeutic options are available^2,6^. Currently, there is a substantial gap in our knowledge of the molecular mechanisms that underpin lung carcinoid biology.

microRNAs (miRNAs) are small (19–24 nt) RNA molecules that typically destabilize multiple messenger RNA (mRNA) molecules through complementary and computationally predictable sequence interactions^7^. These molecules control many cellular processes including cell differentiation and proliferation in neurons and other cells. miRNAs are frequently dysregulated in cancer^8^ and can function as tumor suppressors or oncogenes by targeting genes involved in cell proliferation, survival, apoptosis and metastasis^9,10^. Recently, we found that miR-375 is highly expressed in lung carcinoid tissues^11,12^ and cell lines (unpublished data). Although miR-375 is a promising biomarker for lung and other NENs^11^, its role as a potential lineage oncogene and the underlying mechanism have yet to be explored.

Among many predicted mRNA targets, YAP—a transcriptional co-activator and main effector of the Hippo pathway—is an experimentally validated miR-375 target in lung cancer cell lines^13^. YAP promotes cell proliferation and transformation^14,15^ and YAP overexpression is associated with tumor progression and worse survival in certain malignancies, such as pancreatic cancer^16^. Widely considered to be an oncogene, YAP can also function as a tumor suppressor. Decreased or absent YAP expression is highly correlated with tumor progression and worse survival in breast cancer and other tumors^17^. Thus, YAP has tissue- and/or cell-specific oncogenic or tumor suppressive functions^18,19^. Loss of YAP expression is a characteristic of high-grade NEN cell lines, indicating a potential regulatory role in neuroendocrine differentiation^20,21^. However, the role and mechanism by which YAP regulates neuroendocrine differentiation and tumorigenesis in lung carcinoids remains largely unknown.

Here, we identify and elucidate the mechanism of a miR-375/YAP axis that controls neuroendocrine differentiation and tumorigenesis in a well-differentiated lung carcinoid cell line (H727). We show that miR-375 depletion and YAP overexpression have overlapping transcriptomic changes and similar regulatory effects on neuroendocrine differentiation and tumorigenesis in vitro and in vivo. We also explore the shared downstream RNA targets of this miR-375/YAP axis through pathways analyses, identifying neural differentiation, exocytosis, secretion, and cell cycle pathways that may be dysregulated through disruption of this axis.

## Results

### miR-375 expression in H727 cells is efficiently depleted through CRISPR/Cas9 gene editing

**Figure 1:**
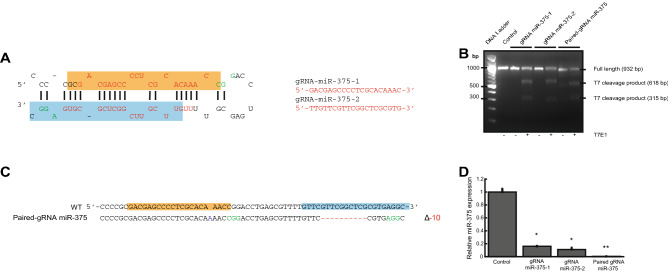
miR-375 expression in H727 cells is efficiently depleted through CRISPR/Cas9 gene editing. (**A**) miR-375 stem-loop. Representation of CRISPR/Cas9 single guide RNAs (gRNAs) targeting Drosha/Dicer processing sites of the miR-375 stem-loop (blue box: mature miR-375/miR-375-3p, orange box: miR-375-5p, red sequence: gRNA and complementary target sequence, green sequence: protospacer adjacent motif). (**B**) T7EI assay. T7 endonuclease (T7EI) cleavage produced the expected DNA bands to confirm CRISPR/Cas9 gene editing. (**C**) Sanger sequencing. Following gene editing, sequencing confirmed the presence of indels within the miR-375 stem-loop sequence (blue box: mature miR-375/miR-375-3p, orange box: miR-375-5p). (**D)** Real-time PCR. miR-375 expression (n = 3) was significantly reduced in miR-375 depleted compared to empty vector control cells (*t* test: *P < 0.05; **P < 0.01). Replicate numbers are indicated (*n*) and all experiments were independently repeated three times.

miR-375 is highly expressed in lung carcinoid tissues^11,12^ and cell lines (Supplementary Fig. 1); miR-375 is the sixth-highest miRNA in H727, contributing approximately 4% of miRNA expression. To investigate miR-375 function in H727 cells, we depleted miR-375 expression through lentiviral CRISPR/Cas9-mediated gene editing (Fig. 1A). Following transduction, we assessed targeting efficiency and specificity using T7EN1-based mismatch detection (Fig. 1B) and DNA sequencing of a 932-bp PCR amplification product encompassing the miR-375 gene locus (Fig. 1C); cleavage products and sequencing indicated the presence of indel mutations adjacent to the protospacer adjacent motif. Lastly, we quantitated miR-375 expression in miR-375-depleted H727 cells using real-time PCR; knockdown efficiency was approx. 98% in paired-gRNA miR-375 H727 cells (Fig. 1D). miR-375-depleted and control H727 cells were used for transcriptomic, functional, and mechanistic studies below.

### miR-375 depletion is associated with distinct transcriptomic and molecular pathway changes in H727 cells

**Figure 2:**
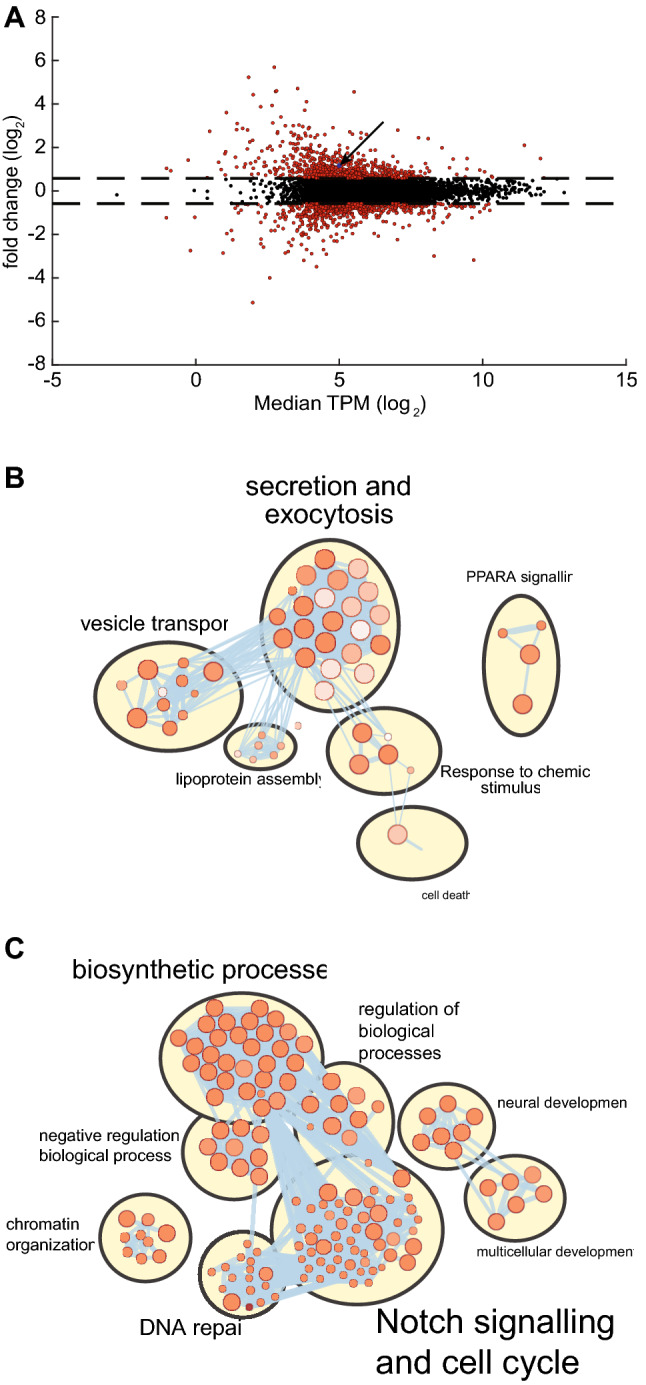
miR-375 depletion is associated with distinct transcriptomic and molecular pathway changes in H727 cells. (**A**) Volcano plot of dysregulated genes between miR-375 depleted (n = 3) and empty vector control cells (n = 3). Following miR-375 depletion, 788 upregulated and 751 downregulated genes were identified. The indicated target gene of miR-375, YAP, was 1.8-fold upregulated. (**B**) Enrichment map of upregulated pathways**.** Of 51 upregulated pathways (indicated as individual bubbles), 22 (43%) pathways were related to secretion and exocytosis. (**C**) Enrichment map of downregulated pathways**.** Of 182 downregulated pathways, 53 pathways (30%) were related to Notch signalling and the cell cycle.

To better understand the RNA regulatory role of miR-375 in H727 cells, we studied transcriptomic changes pre- and post- miR-375 depletion using RNA-seq. Following depletion, we identified 788 upregulated and 751 downregulated genes using a 1.5-fold threshold (Supplementary Table 4, Fig. 2A). Upregulated genes were primarily enriched in pathways related to neuroendocrine functions, secretion and exocytosis, whereas downregulated genes were predominantly enriched in Notch signalling and cell cycle pathways (Supplementary Table 4, Fig. 2B,C). To discover biologically relevant miR-375 targets, we used the Bio-miRTa algorithm^7^ to identify and rank 2677 candidate target genes; YAP was the second highest ranked target (Supplementary Table 5) and was 1.8-fold upregulated following miR-375 depletion. Taken together, miR-375 potentially controls a distinct set of transcripts, including YAP, that mediate functions related to neuroendocrine differentiation and cell proliferation.

### miR-375 depletion reduces neuroendocrine differentiation and suppresses tumorigenesis in vitro and in vivo

**Figure 3:**
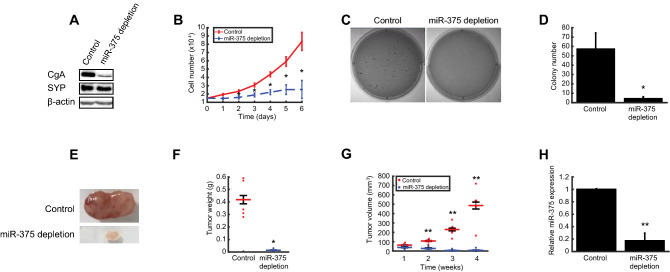
miR-375 depletion reduces neuroendocrine differentiation and suppresses tumorigenesis in H727 cells in vitro and in vivo. (**A**) Western blot analysis of neuroendocrine markers. Blots were cut into two pieces between 50 and 75 kDa, and simultaneously probed with anti-CgA, anti-SYP, or anti β-actin. miR-375 depletion is associated with decreased CgA and SYP expression relative to empty vector control cells. Densitometry analysis provided in Supplementary Fig. 2. (**B**) Cell proliferation. miR-375 depletion significantly reduced cell proliferation (n = 3). Data presented as mean ± SEM (*t* test: *P < 0.05). (**C**,**D**) Colony formation. miR-375 depletion significantly reduced colony formation on soft agar (n = 3). Data presented as mean ± SEM (*t* test: *P < 0.05). (**E**) Images of tumor xenografts. After four weeks, empty vector control (top) or miR-375-depleted (bottom) H727 tumors were harvested. (**F**) Tumor weight. After four weeks, miR-375-depleted xenograft tumors (n = 5) weighed significantly less than tumors derived from empty vector control cells (n = 6) (*t* test: *P < 0.05). (**G**) Tumor volume. miR-375 depleted xenograft tumors (n = 5) were significantly smaller than empty vector control xenograft tumors (n = 6) at weeks 2–4 (*t* test: **P < 0.01). (**H**) miR-375 expression in xenograft tumors. miR-375 expression was significantly lower in xenograft tumors derived from miR-375 depleted (n = 5) compared to empty vector control cells (n = 6) (*t* test: **P < 0.01). Replicate numbers are indicated (*n*) and all experiments were independently repeated three times.

Given that miRNAs regulate a wide range of cellular and disease processes^9^, we assessed the impact of miR-375 depletion on neuroendocrine differentiation and tumorigenesis. Following depletion, we saw substantial and mild reductions of NEN markers CgA and SYP protein levels in H727 cells, respectively (Fig. 3A, Supplementary Fig. 2). Compared to controls, miR-375 depletion was associated with decreased cell proliferation (Fig. 3B) and reduced colony formation (Fig. 3C,D). To investigate the tumorigenic role of miR-375 in vivo, we assessed tumor growth of miR-375-depleted H727 and control cells in a xenograft mouse model. Following four weeks of observation, mice were sacrificed and xenograft tumors removed and evaluated (Fig. 3E). Mean tumor weight and volume were significantly lower for miR-375-depleted than control tumors (Fig. 3F,G). As expected, miR-375 expression was significantly lower in miR-375-depleted than control tumors using real-time PCR (Fig. 3H). CgA and SYP expression levels were also lower in miR-375-depleted than control tumors based on IHC staining (Supplementary Fig. 3). Together, these findings indicate that miR-375 regulates neuroendocrine differentiation, cell growth, and tumorigenesis in H727 cells in vitro and in vivo.

### YAP overexpression is associated with distinct transcriptomic and molecular pathway changes in H727 cells

**Figure 4:**
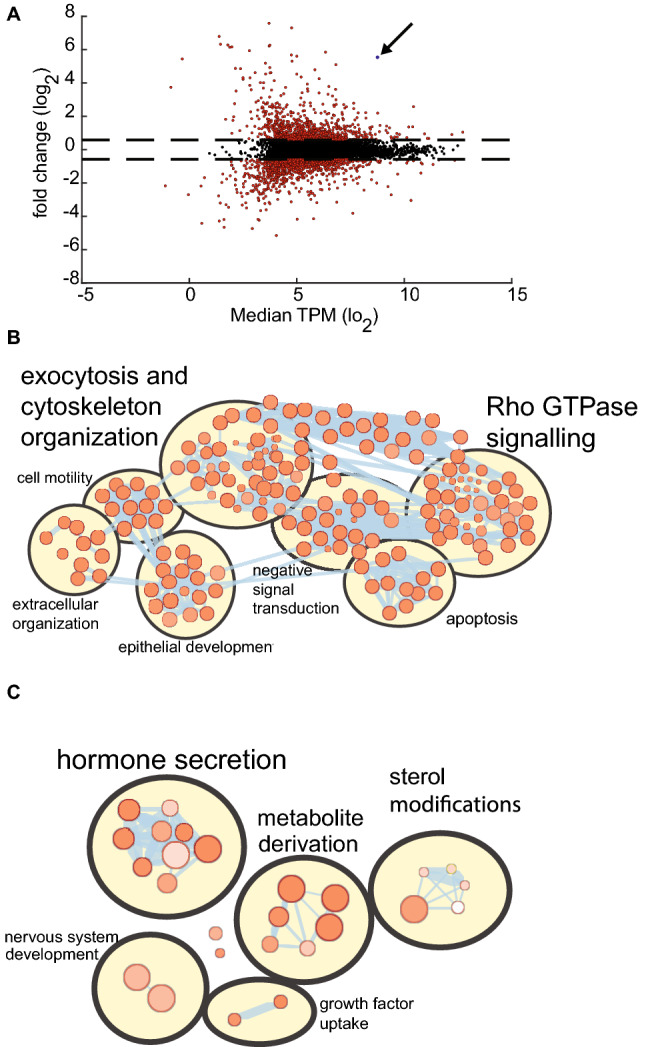
YAP overexpression is associated with distinct transcriptomic and molecular pathway changes in H727 cells. (**A**) Volcano plot of dysregulated genes between Dox-induced YAP overexpression (n = 3) and control cells (n = 3). Following YAP overexpression, 937 upregulated and 1032 downregulated genes were identified. YAP (indicated) was 46.6-fold upregulated. (**B**) Enrichment map of upregulated pathways**.** Of 237 upregulated pathways (indicated as individual bubbles), 48 pathways (20%) were related to exocytosis and cytoskeletal organization. (**C**) Enrichment map of downregulated pathways**.** Of 26 downregulated pathways, 9 pathways (35%) were related to hormone secretion.

To identify biologically relevant miR-375 targets we used the Bio-miRTa algorithm^7^, which ranked YAP as the second highest predicted gene target (Supplementary Table 5). We confirmed that YAP is a *bona fide* target of miR-375 using a luciferase reporter assay (Supplementary Fig. 4), in agreement with previous studies^13^. Subsequently, we reasoned that YAP overexpression should have similar transcriptomic and functional consequences as miR-375 depletion in H727 cells. To better understand the RNA regulatory role of YAP in H727 cells, we studied transcriptomic changes pre- and post- YAP overexpression using RNA-seq. Following overexpression, we identified 937 upregulated and 1032 downregulated genes using a 1.5-fold threshold (Supplementary Table 6, Fig. 4A). Upregulated genes were primarily enriched in exocytosis and cytoskeletal organization pathways whereas downregulated genes were predominantly enriched in hormone secretion pathways (Supplementary Table 6, Fig. 4B,C). Taken together, YAP potentially controls a distinct set of transcripts that mediate functions related to neuroendocrine differentiation and cell proliferation, in part resembling pathways dysregulated by miR-375 depletion.

### YAP overexpression in H727 cells phenocopies miR-375 depletion in vitro and in vivo

**Figure 5:**
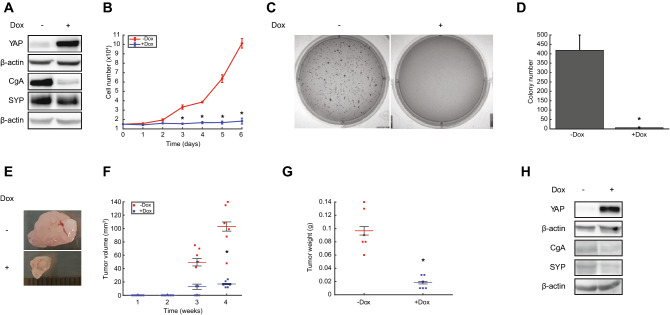
YAP overexpression reduces neuroendocrine differentiation and suppresses tumorigenesis in H727 cells in vitro and in vivo. (**A**) Western blot analysis of neuroendocrine markers. Blots were cut into two pieces between 50 and 75 kDa, and simultaneously probed with anti-CgA, anti-SYP, or anti β-actin. Dox-induced YAP overexpression resulted in decreased expression of CgA and SYP. Densitometry analysis provided in Supplementary Fig. 5. (**B**) Cell proliferation. Dox-induced YAP overexpression significantly reduced cell proliferation (n = 3). Data presented as mean ± SEM (*t* test: *P < 0.05). (**C**,**D**) Colony formation. Dox-induced YAP overexpression significantly suppressed colony formation on soft agar (n = 3). Data presented as mean ± SEM (*t* test: *P < 0.05). (**E**) Images of tumor xenografts. H727-YAP-S127A cells without (−) or with (+) Dox feed after four weeks of tumor growth. (**F**) Tumor volume. H727-YAP-S127A (+) Dox (n = 6) were significantly smaller than (−) Dox xenograft tumors (n = 6) at week 4 (*t* test: *P < 0.05). (**G**) Tumor weight. After four weeks, H727-YAP-S127A (+) Dox xenograft tumors (n = 6) weighed significantly less than (−) Dox xenograft tumors (n = 6) (*t* test: *P < 0.05). (**H**) Western blot analysis of YAP and neuroendocrine marker (CgA and SYP) expression in xenograft tumors. Blots were cut into two pieces between 50 and 75 kDa, and simultaneously probed with anti-YAP, anti-CgA, anti-SYP, or anti β-actin. YAP expression was increased and CgA and SYP expression decreased in H727-YAP-S127A (+) Dox compared to (−) Dox xenograft tumors. Densitometry analysis provided in Supplementary Fig. 6. Replicate numbers are indicated (*n*) and all experiments were independently repeated three times.

To assess phenotypic similarities between YAP overexpression and miR-375 depletion, we overexpressed constitutively active YAP (YAP-S127A), in which LATS1/2 kinase phosphorylation/inactivating site Serine (S) 127 is mutated into alanine (A), in H727 cells and examined its effects on neuroendocrine differentiation and tumorigenesis in vitro and in vivo. Following Dox treatment, we saw substantial and mild reductions of CgA and SYP protein levels in H727 cells, respectively (Fig. 5A, Supplementary Fig. 5). Compared to controls, YAP overexpression was associated with decreased cell proliferation (Fig. 5B) and reduced colony formation (Fig. 5C,D). To investigate the tumorigenic role of YAP in vivo, we assessed tumor growth of YAP overexpression and control cells in a mouse xenograft model. Following four weeks of observation, mice were sacrificed and xenograft tumors removed and evaluated (Fig. 5E). Similar to miR-375 depletion, mean tumor weight and volume were significantly lower for YAP overexpression than control tumors (Fig. 5F,G). As expected, higher YAP and lower CgA and SYP expression was detected in YAP overexpression than control tumors using WB (Fig. 5H, Supplementary Fig. 6). CgA and SYP expression levels were also lower in YAP overexpression than control tumors based on IHC analyses (Supplementary Fig. 7). With similar effects on neuroendocrine differentiation, growth, and tumorigenesis, these findings indicate that YAP overexpression phenocopies miR-375 depletion in H727 cells.

### Evidence for and mechanistic implications of a miR-375/YAP axis

**Figure 6:**
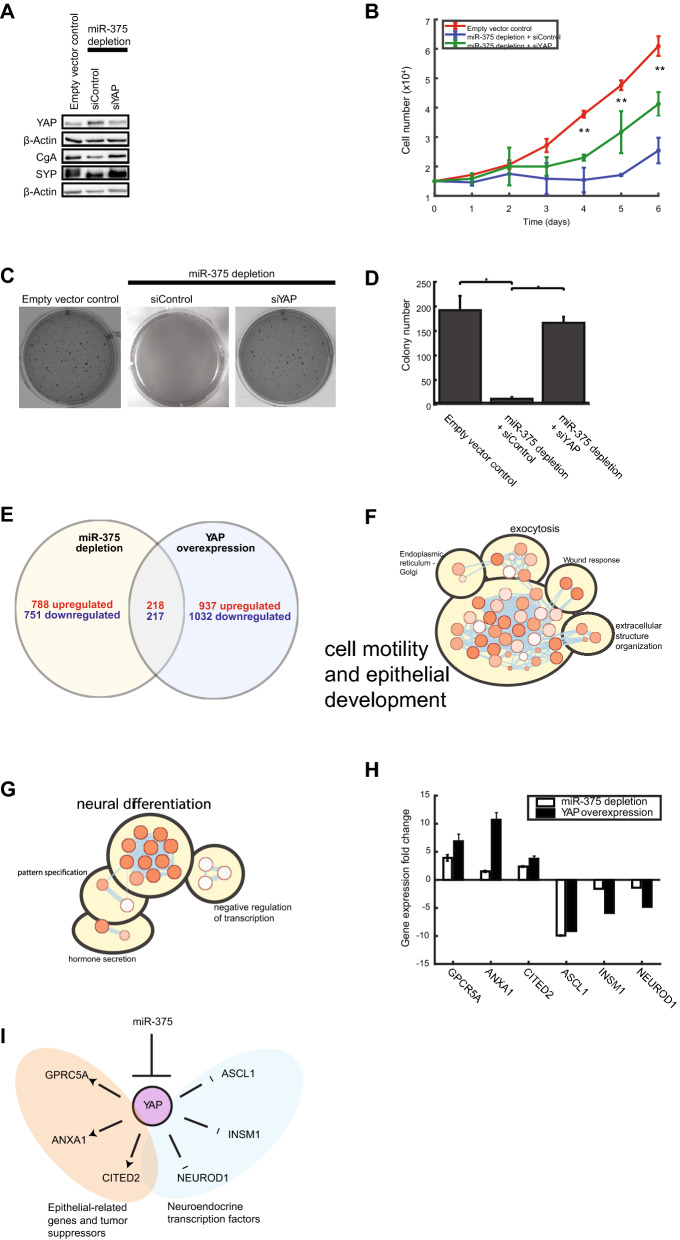
Evidence for and mechanistic implications of a miR-375/YAP axis. (**A**) Neuroendocrine marker expression after YAP knockdown. Blots were cut into two pieces between 50 and 75 kDa, and simultaneously probed with anti-CgA, anti-SYP, or anti β-actin. Following siYAP transfection of miR-375-depleted cells, CgA and SYP expression was recovered. (**B**) Cell proliferation after YAP knockdown. Following siYAP transfection of miR-375-depleted cells, cell proliferation was partially recovered (n = 3) (ANOVA test: **P < 0.01). (**C**,**D**) Soft agar assay after YAP knockdown. Following siYAP transfection of miR-375-depleted cells, colony formation was recovered (n = 3) (*t* test: **P < 0.01). (**E**) Venn diagram of miR-375/YAP dysregulated genes. Dysregulated gene sets from miR-375 depletion and YAP overexpression were overlapped to define the shared gene set: 218 genes were upregulated and 217 genes were downregulated by the miR-375/YAP axis. (**F**) Enrichment map of upregulated pathways**.** Of 46 upregulated pathways mediated by the miR-375/YAP axis, 33 pathways (72%) were related to cell motility and epithelial development. (**G**) Enrichment map of downregulated pathways**.** Of 18 downregulated pathways mediated by the miR-375/YAP axis, 13 pathways (72%) were related to neural differentiation and hormone secretion. (**H**) Real-time PCR. Gene expression fold change of select neuroendocrine transcription factors, oncogenes or tumor suppressor genes dysregulated by miR-375/YAP (n = 3; mean ± SEM). (**I**) miR-375/YAP axis. Schematic of proposed miR-375/YAP axis mediating neuroendocrine differentiation and tumorigenesis through direct or indirect regulation of downstream genes. Replicate numbers are indicated (*n*) and all experiments were independently repeated three times.

Based on the similarities above, we hypothesized that a miR-375/YAP axis controls neuroendocrine differentiation and tumorigenesis in H727 cells. To test this hypothesis, we targeted YAP in miR-375-depleted H727 cells using an siRNA approach. YAP knockdown increased CgA and SYP expression (Fig. 6A), partially rescued cell growth inhibition caused by miR-375 depletion (Fig. 6B), recovered colony formation in soft agar (Fig. 6C,D) and the expression of some neuroendocrine transcription factors (Supplementary Fig. 8). Thus, a miR-375/YAP axis mediates neuroendocrine cell differentiation, proliferation and tumorigenesis in H727 cells.

To further explore the mechanistic implications of this axis, we identified 435 genes commonly dysregulated in miR-375-depleted and YAP overexpression cells (Fig. 6E). Pathways analysis showed that genes upregulated by the axis (n = 218) were primarily involved in cell motility and epithelial development (Supplementary Table 7, Fig. 6F) whereas genes downregulated by the axis (n = 217) were involved in neural differentiation and hormone secretion, among other functions (Supplementary Table 7, Fig. 6G). A subset of upregulated (GPRC5A, ANXA1, CITED2) and downregulated (ASCL1, INSM1, NEUROD1) genes were analyzed by SYBR Green qRT-PCR, confirming that these genes were increased or decreased after miR-375 depletion or YAP overexpression (Fig. 6H). Further, we confirmed these patterns in gene expression data from human lung carcinoid tissue^22^ (Supplementary Fig. 9). Our results indicate that the miR-375/YAP axis controls neuroendocrine differentiation and tumorigenesis through direct or indirect regulation of mechanistically relevant genes, summarized in Fig. 6I.

## Discussion

Although miR-375 is highly expressed in lung carcinoid tissues and cell lines, its RNA regulatory and functional roles remain to be determined. Here, we show that miR-375 targets a distinct set of transcripts, including *YAP*, and is a potent regulator of neuroendocrine differentiation and tumorigenesis. As expected for a *bona fide* miRNA target, YAP overexpression phenocopies miR-375 depletion. Lastly, we show that miR-375 and YAP form a molecular axis, through which well-known downstream genes related to neuroendocrine phenotype and tumorigenesis are potentially regulated. Our study provides a better understanding of RNA regulatory networks and molecular mechanisms in lung carcinoids that can be leveraged for diagnostic and therapeutic purposes.

In the present study, we depleted miR-375 in H727 lung carcinoid cells using a lentivirus CRISPR/Cas9-mediated strategy. Following depletion, transcriptomic and pathway analyses indicated that genes associated with secretory and exocytotic functions are upregulated whereas those associated with Notch signalling and cell cycle regulation are downregulated. Functional studies of miR-375-depleted cells showed decreased neuroendocrine differentiation, substantial inhibition of cell proliferation and decreased anchorage-independent colony formation, and considerably slower tumor growth in xenograft models. miR-375 is known to regulate lineage-specific differentiation of lung neuroendocrine cancer cells^13^, but its role in RNA regulation and tumorigenesis in carcinoids is unclear. Although considered a tumor suppressor in many cancers^23^, the high abundance of miR-375 in lung carcinoids argues for an oncogenic or bystander role; our experimental data indicate that miR-375 is a novel oncogene in H727 cells.

YAP is one of hundreds of computationally predicted miR-375 targets. Based on its second ranking in Bio-miRTa analysis and our confirmation of its known targeting ability^13^, we decided to study its transcriptional impact and functional roles in lung carcinoid cells. Accordingly, we used a lentivirus-mediated approach to overexpress YAP in H727 cells, uncovering similar transcriptomic and functional changes as seen with miR-375 depletion. Following YAP overexpression, transcriptomic and pathway analyses indicated that genes associated with exocytosis and cytoskeletal organization functions are upregulated whereas those associated with hormone secretion are downregulated. Functional studies of YAP overexpression cells showed reduced neuroendocrine differentiation, inhibition of cell proliferation and decreased anchorage-independent colony formation, and slower tumor growth in xenograft models. YAP was also recently shown to be a critical regulator of neuroendocrine differentiation in high-grade neuroendocrine lung tumors; YAP loss correlated with strong expression of neuroendocrine markers^20^. Similar to miR-375, YAP has oncogenic and tumor-suppressive activities in different cancers^17–19^. Our experimental data indicate that YAP is a tumor suppressor in H727 cells and that YAP overexpression phenocopies miR-375 depletion.

We found strong evidence for a functional miR-375/YAP axis in H727 cells. YAP knockdown in miR-375-depleted cells recovered neuroendocrine marker expression, partially rescued cell growth and rescued colony formation in soft agar. Given that YAP acts as a tumor suppressor in some cancers^18^, we expect that sustained YAP loss in the context of miR-375 depletion would also recover in vivo tumor growth. To further explore this molecular axis and gain downstream mechanistic insights, we identified 435 dysregulated genes shared between miR-375 depletion and YAP overexpression states. Of these, upregulated genes were enriched in cell motility and epithelial development pathways and downregulated genes were enriched in neural differentiation pathways. Established target genes of YAP, such as connective tissue growth factor (CTGF) and cysteine-rich protein 61 (Cyr61), were not identified among these commonly dysregulated genes. Instead, epithelial-related genes (GPRC5A and CITED2) and tumor suppressors (GPRC5A and ANXA1) were upregulated and neuroendocrine transcription factors (ASCL1, NEUROD1 and INSM1, among others) were downregulated. GPRC5A positively modulates epithelial cell adhesion^24^ and is a documented tumor suppressor in lung cancer cells^25^, ANXA1 is a tumor suppressor that inhibits the NF-KB pathway^26^, and CITED2 is a transcriptional modulator that controls differentiation of lung epithelial cells^27^. ASCL1 induces neuroendocrine differentiation^28^ and also regulates multiple genes involved in cell cycle progression, including canonical cell cycle regulators and oncogenic transcription factors^29^. Similarly, over-expression of NEUROD1 in non-neuroendocrine lung cancer cell lines is sufficient to increase cell proliferation and activate a neuroendocrine program^30^. ASCL1 and NEUROD1 distinguish heterogeneity in SCLC with distinct genomic landscapes and gene expression programs^31^, and INSM1 regulates neuroendocrine differentiation in lung cells^32^, directs transcription^13,32,33^ and inhibits cell cycle progression by binding to cyclin D1^34^. Given the function of these dysregulated genes, we propose that the miR-375/YAP axis is a dominant switch in neuroendocrine differentiation and tumorigenesis through control of lineage-defining genes and transcription factors (Fig. 6G).

Our study showed that YAP knockdown only partially rescued cell growth changes associated with miR-375 depletion and did not assess the impact of miR-375/YAP on metastasis or negative feedback in the Hippo pathway. In addition to YAP, Bio-miRTa target prediction found other potentially relevant miR-375 targets, including PLEHKA3, with changes in gene expression following miR-375 depletion (data not shown). Therefore, other miR-375 targets may mediate miR-375-induced neuroendocrine differentiation and tumorigenesis. Nonetheless, our data show that YAP is an important component of these phenotypic changes. We expect that miR-375/YAP will have a similar effect on metastasis as cell proliferation, given that YAP acts as a tumor suppressor and inhibitor of metastasis in other cancer cells^35^. However, repression of metastasis through miR-375/YAP should be validated in the context of lung carcinoids with invasion and migration assays. The role of miR-375/YAP in Hippo pathway negative feedback also requires further investigation; activated YAP normally induces transcription of LATS2, but LATS2 may be post-translationally modified in cancer cells^36^, like lung carcinoids, to prevent negative feedback. In summary, we have demonstrated the phenotypic importance of miR-375/YAP in lung carcinoid cells and highlighted directions for future studies.

Our findings indicate that a miR-375/YAP axis has a critical regulatory role in neuroendocrine differentiation and tumorigenesis of lung carcinoid cells in vitro and in vivo. This axis can be leveraged for diagnostic and therapeutic purposes, and screening of downstream genes in this axis is expected to provide more complete elucidation of the molecular networks underpinning neuroendocrine differentiation and tumorigenesis in lung carcinoids.

## Materials and methods

### Cell lines and cell culture

Lung carcinoid (NCI-H727) and human embryonic kidney (HEK) 293T cell lines were obtained from the American Type Culture Collection (ATCC). H727 cells were grown in ATCC-formulated RPMI-1640 (Invitrogen) supplemented with 10% fetal bovine serum (FBS). HEK 293T cells were cultured in Dulbecco’s modified Eagle’s medium (DMEM; Sigma-Aldrich) supplemented with 10% FBS, and 1% penicillin/streptomycin (Invitrogen). Both lines were maintained at 37 °C in a humidified 5% CO_2_ incubator.

### Plasmid construction

Transfer vectors enabling lentivirus-mediated miR-375 depletion (LentiCRISPR-gRNAs-miR-375) or constitutively active YAP expression (pTRIPZ-YAP-S127A) were generated as described below or as previously reported^37^. Briefly, lentiCRISPR v1 (Addgene; plasmid #49353) vector DNA was digested using FastDigest BsmBI (Fermentas) and FastAP Alkaline Phosphatase (Fermentas) prior to purification with the QIAquick Gel Extraction Kit (Qiagen). Guide RNA (gRNA) sequences targeting the Drosha or Dicer processing sites in the human miR-375 precursor sequence were designed using the CRISPR DESIGN (http://crispr.mit.edu/) web tool (Supplementary Table 1) and synthesized as complementary single-stranded oligonucleotides with BsmBI overhangs (Life Technologies). Following phosphorylation with T4 polynucleotide kinase (NEB), oligonucleotides were annealed using the following thermal cycling conditions: 37 °C for 30 min and 90 °C for 5 min with a stepwise reduction in temperature to 25 °C at a rate of − 5 °C/min. Annealed oligonucleotides were ligated into digested lentiCRISPR v1 vector using the Quick Ligation Kit (NEB) and the resulting plasmid LentiCRISPR-gRNAs-miR-375 was transformed into Stbl3 bacteria (propagated from Invitrogen C7373-03).

### Lentivirus production and cell transduction

To produce lentivirus, HEK 293T cells seeded in 100-mm plates were transfected with 2.5 μg expression plasmid (lentiCRISPR-gRNAs-miR-375 or pTRIPZ-YAP-S127A), 1.875 μg packaging plasmid (psPAX2), and 0.626 μg envelope plasmid (pMD2G) using PolyJet reagent (SignaGen Laboratories) according to the manufacturer’s instructions. Cells were subsequently grown in DMEM (high glucose) containing 10% FBS for 48 h. Lentivirus-containing supernatants were harvested, passed through a 0.45-μm filter (Sarstedt), and either used immediately or flash frozen in liquid nitrogen and stored at − 80 °C.

Stable miR-375-depleted H727 or doxycycline (Dox)-inducible YAP overexpression H727 (H727-YAP-S127A) cells were established by infection with lentivirus and subsequent antibiotic selection. Briefly, H727 cells were grown to 30–50% confluence in 6-well plates prior to replacement of the complete growth medium (ATCC-formulated RPMI-1640 containing 10% FBS) with virus solution containing 8 μg/mL Polybrene (Sigma Aldrich). Following an incubation period of 12 h at 37 °C, virus-containing medium was refreshed and cells further cultured for 24 h. Stable miR-375-depleted H727 or H727-YAP-S127A cells were respectively selected with 2 μg/mL puromycin or 800 μg/mL hygromycin over a period of 8–9 days. Finally, single cell clones were obtained for miR-375-depleted H727 by limiting dilution and expansion. Once established, stable cells were used for molecular characterization or in functional assays below; H727-YAP-S127A cells were treated with 1 µg/mL doxycycline hyclate (Bioshop Canada) for 48 h prior to use.

### Verification of CRISPR-mediated genome editing

CRISPR-mediated genome editing was verified by T7 endonuclease I (T7E1) mismatch detection assay and Sanger sequencing. For mismatch verification, genomic DNA from miR-375-depleted and empty vector control H727 cells was extracted using the PureLink Genomic DNA Mini Kit (Thermo Fisher Scientific). A 932-bp PCR amplification product, spanning the expected editing sites, was generated using PrimeSTAR GXL DNA Polymerase (Clontech) and custom primers (Supplementary Table 2) prior to purification with the QIAquick PCR Purification Kit (Qiagen). T7EN1 (NEB) mismatch detection assay was performed according to manufacturer’s instructions. Mismatched products were separated by 1% agarose gel electrophoresis containing 0.5 μg/mL ethidium bromide and images acquired using an Amersham Imager 600 (GE Healthcare Life Science). For sequencing-based verification, the PCR product subjected to T7EN1 enzyme digestion was also ligated into pGEM-T Easy vectors (Promega) for Sanger sequencing at the McGill University and the Génome Québec Innovation Centre.

### RNA isolation and quality control

Total RNA was isolated from miR-375-depleted, YAP overexpression, and control cells using TRIzol Reagent (Invitrogen) according to manufacturer’s instructions. Total RNA concentration and purity was determined using a SmartSpec™ Plus Spectrophotometer (Bio-Rad Laboratories) and 1% agarose gel electrophoresis.

### RNA sequencing and annotation

miRNA and mRNA expression profiles were generated in triplicate for miR-375-depleted, YAP overexpression, and control cells using small RNA sequencing and RNA-seq. miRNA profiles were generated using a well-established sequencing, annotation, and analysis pipeline^38,39^. RNA-seq libraries were prepared using the NEBNext Ultra RNA Library Prep Kit (NEB, E7530) and the NEBNext rRNA depletion kit (NEB, E6310) according to the manufacturer’s instructions; RNA fragmentation was performed for 10 min. Following sequencing on the Illumina HiSeq 3000 platform, FASTQ sequence files were de-multiplexed prior to annotation and downstream analyses. Raw sequence reads were assessed for quality using FastQC (v.0.11.8)^40^ and adapter sequences and low-quality reads removed using Trimmomatic (v.0.36)^41^. Reads were annotated against the human reference transcriptome GRCh38 (release 97) using an established alignment and quantification tool (Kallisto v.0.46.0)^42^. Sequencing data reported in this paper have been deposited in NCBI’s Gene Expression Omnibus and are accessible through GEO Series GSE154872.

### Quantitative miRNA and mRNA real-time PCR

Select miRNA and mRNA targets were respectively measured in triplicate and in duplicate using reverse transcription quantitative real-time PCR (real-time PCR). miR-375 and endogenous RNU6B were quantitated using TaqMan MicroRNA Assays (Applied Biosystems, Assay IDs: 000564, 001093) as described^43^; relative miRNA expression was calculated using the ΔCt method^44^. Following bulk first-strand cDNA synthesis using the SuperScript III First-Strand Synthesis System (Invitrogen), mRNAs of interest and 18S rRNA were quantitated on a ViiA 7 Real-Time PCR System (Thermo Fisher Scientific); each reaction contained cDNA from 25 ng of total RNA, 2 × QuantiFast SYBR Green PCR Master Mix (Qiagen), and gene-specific primers (Supplementary Table 3) and amplified using the following thermal cycling conditions: 95 °C for 5 min followed by 40 cycles of 95 °C for 10 s and 60 °C for 30 s. Mean and SEM Ct values for each target were obtained and relative mRNA expression calculated using the 2^−ΔΔCt^ method^44^.

### siRNA-mediated gene expression knockdown

siRNA duplexes targeting YAP (siYAP) and an siRNA with scrambled sequence (siControl) were purchased from Integrated DNA Technologies. miR-375-depleted H727 cells were transfected with 50 nmol/L of siRNAs using GenMute siRNA and DNA Transfection Reagent (SignaGen Laboratories) according to the manufacturer’s instructions. Forty-eight hours post transfection, cells were collected and proteins extracted for Western blot analysis.

### Western blot analysis

Cells were rinsed three times with ice-cold 1 × PBS, scraped in RIPA buffer [150 mM NaCl, 50 mM Tris–HCl (pH 7.4), 5 mM EDTA (pH 8.0),1% NP-40, 0.5% sodium deoxycholate, 0.1% sodium dodecyl sulfate (SDS)] containing 1 × Halt Protease Inhibitor Cocktail (Thermo Scientific), agitated for 30 min at 4 °C, and centrifuged at 12,000 rpm for 20 min. Protein concentrations were quantified using the BCA Protein Assay Kit (Thermo Fisher Scientific). For western blot analysis, protein samples were mixed with 6 × SDS sample buffer (375 mM Tris–HCl, 9% SDS, 50% glycerol, 9% beta-mercaptoethanol, 0.03% bromophenol blue), boiled for 5 min, separated by SDS-PAGE, and electrophoretically transferred to nitrocellulose membrane (BIO-RAD Laboratories). After blocking [5% nonfat milk and TBST (0.1% Tween 20 in Tris-buffered saline containing 50 mM Tris and 150 mM NaCl, pH 7.5)] for 1 h, blots were cut into two pieces between 50–75 kDa and probed with mouse monoclonal [LK2H10] anti-chromogranin A (Abcam, ab8204, 1:100), rabbit monoclonal [YE269] anti-synaptophysin (Abcam, ab32127, 1:20,000), mouse monoclonal anti-YAP (Santa Cruz, sc-101199, 1:200), or mouse monoclonal anti-β-actin antibody (Sigma, A5441, 1:10,000) in blocking buffer overnight at 4 °C. Blots were washed three times in TBST for 5 min, incubated with peroxidase-labeled anti-mouse IgG antibody (Abcam, ab6789, 1:3000) or anti-rabbit IgG antibody (Abcam, ab6721, 1:3000) in blocking buffer for 1 h, washed as above, detected using Clarity Western Enhanced Chemiluminescence Substrate (BIO-RAD Laboratories), and visualized using an Amersham Imager 600UV.

### Cell proliferation assay

Cell proliferation assays were performed as described^45^ and repeated at least three times. Briefly, 1.5 × 10^4^ cells from each transduced cell line were seeded into each well of a 12-well plate in triplicate and counted daily for 6 days; YAP expression was induced on alternate days using fresh culture medium containing doxycycline (1 μg/mL).

### Soft agar colony formation assay

Soft agar colony formation was assessed as described^45^ and repeated at least three times. Briefly, 2 × 10^4^ cells from each transduced cell line were mixed with complete growth medium containing 0.4% agarose and overlaid on 0.8% agarose in each well of a 6-well plate in triplicate; YAP expression was induced on alternating days as above. Following an incubation period of 18 days in a CO_2_ incubator (5% CO_2_, 37 °C), colonies were stained with crystal violet (0.005% crystal violet in 20% methanol), imaged using a Bio-Rad Gel Doc System (Bio-Rad Laboratories), and quantified using the colony count program in the Quantity One software package.

### Xenograft mouse models

Xenograft mouse models were used to assess differences in in vivo tumor formation between experimental (miR-375 depleted or YAP overexpression) and control cells. Based on previous sample size calculations (effect size: 1.8 $\sigma$, $\alpha$: 0.05, power: 0.8)^46^, six or twelve mice were used per experimental group. Mice were randomly selected based on age from available colony litters with balanced M:F ratios. Parental H727 cells were injected to control for any confounding effects of Dox treatment. Personnel were initially blinded to group allocation, but blinding was not maintained due to obvious differences in tumor growth. Briefly, 1 × 10^6^ experimental or control cells were suspended in 100 μL (1:1 dilution) of cold 1 × PBS and Growth Factor Reduced, Phenol-Red Free Matrigel (BD Bioscience) and injected subcutaneously into opposing flanks of six or twelve nude mice (12-week-old Rag2/IL2Rgc double knockout mice) for miR-375-depleted and H727-YAP-S127A studies, respectively; H727-YAP-S127A and control mice were fed ad libitum with either Dox-containing (#C14100-0625I, Cedarlane, Canada) or normal chow (#5021, Lab Diet, Purina USA) as indicated. Mice were subsequently assessed at weekly intervals with serial measurements of weight and palpable tumor size; tumor volume was calculated as length x width^2^ × 0.5. Mice were euthanized at four weeks. Harvested tumors were either fixed in formalin and embedded in paraffin wax or flash frozen in liquid nitrogen for subsequent molecular studies. Due to technical error at the time of injection, one miR-375-depleted H727 data point was excluded from analysis. All procedures were approved by the Queen's University Animal Care Committee in accordance with the Canadian Council on Animal Care guidelines and in compliance with the ARRIVE guidelines.

### Statistical analyses

Two-sided student’s *t* test was used for comparisons between experimental and control conditions. Experiments containing more than two experimental groups were evaluated using the ANOVA test. A *P* value of < 0.05 was regarded as statistically significant, P-values are indicated in the figures as: *P < 0.05, **P < 0.01.

### Bioinformatic analyses

Differentially expressed mRNAs were identified by comparing median gene expression (transcripts per million; TPM) between miR-375 depleted, YAP overexpression, and control cells; differentially expressed genes met a threshold 1.5-fold change in expression. Pathways analysis was performed on sets of differentially expressed genes using gProfiler^47^ with GO biological processes^48,49^ and Reactome^50^ databases. Enriched pathways were visualized in Cytoscape (v.3.7.0)^51^ using EnrichmentMap^52^ and Autoannotate^53^. Candidate miR-375 targets were identified and ranked using the Bio-miRTa target prediction algorithm^7^. Briefly, in the first step of Bio-miRTa, the algorithm extracted and ranked miRNA-375 targets from 6 databases (4 target prediction algorithms; TargetScan, miRanda, DIANA and PITA, and 2 experimentally validated targets; TarBase and starBase). In the second step, the algorithm used differential gene expression analysis between control and miR-375 depleted H727 cells for the final ranking of miR-375 predicted gene targets.

## Supplementary Information

Supplementary Information 1.

Supplementary Information 2.

Supplementary Information 3.

Supplementary Table 4.

Supplementary Table 5.

Supplementary Table 6.

Supplementary Table 7.

## Data Availability

RNA-sequencing data have been submitted to GEO (GSE154872) and are available to reviewers (https://www.ncbi.nlm.nih.gov/geo/query/acc.cgi?acc=GSE154872) using the private token: mhafywsohdwblsb.

## References

[1] Hendifar AE, Marchevsky AM, Tuli R (2017). Neuroendocrine tumors of the lung: Current challenges and advances in the diagnosis and management of well-differentiated disease. J. Thorac. Oncol..

[2] Derks JL, Leblay N, Lantuejoul S, Dingemans AC, Speel EM, Fernandez-Cuesta L (2018). New insights into the molecular characteristics of pulmonary carcinoids and large cell neuroendocrine carcinomas, and the impact on their clinical management. J. Thorac. Oncol..

[3] Hilal T (2017). Current understanding and approach to well differentiated lung neuroendocrine tumors: An update on classification and management. Ther. Adv. Med. Oncol..

[4] Swarts DRA, Ramaekersa FCS, Speel EJM (2015). Gene expression profiling of pulmonary neuroendocrine neoplasms: A comprehensive overview. Cancer Treat. Commun..

[5] Robelin P, Hadoux J, Forestier J, Planchard D, Hervieu V, Berdelou A, Scoazec JY, Valette PJ, Leboulleux S, Ducreux M (2019). Characterization, prognosis, and treatment of patients with metastatic lung carcinoid tumors. J. Thorac. Oncol..

[6] Torniai M, Scortichini L, Tronconi F, Rubini C, Morgese F, Rinaldi S, Mazzanti P, Berardi R (2019). Systemic treatment for lung carcinoids: From bench to bedside. Clin. Transl. Med..

[7] Michael IP, Saghafinia S, Hanahan D (2019). A set of microRNAs coordinately controls tumorigenesis, invasion, and metastasis. Proc. Natl. Acad. Sci. U.S.A..

[8] Lu J, Getz G, Miska EA, Alvarez-Saavedra E, Lamb J, Peck D, Sweet-Cordero A, Ebert BL, Mak RH, Ferrando AA (2005). MicroRNA expression profiles classify human cancers. Nature.

[9] Peng Y, Croce CM (2016). The role of MicroRNAs in human cancer. Signal Transduct. Target Ther..

[10] Farazi TA, Hoell JI, Morozov P, Tuschl T (2013). MicroRNAs in human cancer. Adv. Exp. Med. Biol..

[11] Nanayakkara J, Tyryshkin K, Yang X, Wong JJM, Vanderbeck K, Ginter PS, Scognamiglio T, Chen Y, Panarelli N, Cheung N (2020). Characterizing and classifying neuroendocrine neoplasms through microRNA sequencing and data mining. NAR Cancer.

[12] Wong JJM, Ginter PS, Tyryshkin K, Yang X, Nanayakkara J, Zhou Z, Tuschl T, Chen Y, Renwick N (2020). Classifying lung neuroendocrine neoplasms through microRNA Sequence Data Mining. Cancers (Basel).

[13] Nishikawa E, Osada H, Okazaki Y, Arima C, Tomida S, Tatematsu Y, Taguchi A, Shimada Y, Yanagisawa K, Yatabe Y (2011). miR-375 is activated by ASH1 and inhibits YAP1 in a lineage-dependent manner in lung cancer. Cancer Res..

[14] Zanconato F, Cordenonsi M, Piccolo S (2016). YAP/TAZ at the roots of cancer. Cancer Cell.

[15] Lo Sardo F, Strano S, Blandino G (2018). YAP and TAZ in lung cancer: Oncogenic role and clinical targeting. Cancers (Basel).

[16] Rozengurt E, Sinnett-Smith J, Eibl G (2018). Yes-associated protein (YAP) in pancreatic cancer: At the epicenter of a targetable signaling network associated with patient survival. Signal Transduct. Target Ther..

[17] Yuan M, Tomlinson V, Lara R, Holliday D, Chelala C, Harada T, Gangeswaran R, Manson-Bishop C, Smith P, Danovi SA (2008). Yes-associated protein (YAP) functions as a tumor suppressor in breast. Cell Death Differ..

[18] Wang H, Du YC, Zhou XJ, Liu H, Tang SC (2014). The dual functions of YAP-1 to promote and inhibit cell growth in human malignancy. Cancer Metastasis Rev..

[19] Zhang X, Abdelrahman A, Vollmar B, Zechner D (2018). The ambivalent function of YAP in apoptosis and cancer. Int. J. Mol. Sci..

[20] Ito T, Matsubara D, Tanaka I, Makiya K, Tanei ZI, Kumagai Y, Shiu SJ, Nakaoka HJ, Ishikawa S, Isagawa T (2016). Loss of YAP1 defines neuroendocrine differentiation of lung tumors. Cancer Sci..

[21] Horie M, Saito A, Ohshima M, Suzuki HI, Nagase T (2016). YAP and TAZ modulate cell phenotype in a subset of small cell lung cancer. Cancer Sci..

[22] Laddha SV, Da Silva EM, Robzyk K, Untch BR, Ke H, Rekhtman N, Poirier JT, Travis WD, Tang LH, Chan CS (2019). Integrative genomic characterization identifies molecular subtypes of lung carcinoids. Cancer Res..

[23] Yan JW, Lin JS, He XX (2014). The emerging role of miR-375 in cancer. Int. J. Cancer.

[24] Bulanova DR, Akimov YA, Rokka A, Laajala TD, Aittokallio T, Kouvonen P, Pellinen T, Kuznetsov SG (2017). Orphan G protein-coupled receptor GPRC5A modulates integrin beta1-mediated epithelial cell adhesion. Cell Adhes. Migr..

[25] Tao Q, Fujimoto J, Men T, Ye X, Deng J, Lacroix L, Clifford JL, Mao L, Van Pelt CS, Lee JJ (2007). Identification of the retinoic acid-inducible Gprc5a as a new lung tumor suppressor gene. J. Natl. Cancer Inst..

[26] Zhang Z, Huang L, Zhao W, Rigas B (2010). Annexin 1 induced by anti-inflammatory drugs binds to NF-kappaB and inhibits its activation: Anticancer effects in vitro and in vivo. Cancer Res..

[27] Xu B, Qu X, Gu S, Doughman YQ, Watanabe M, Dunwoodie SL, Yang YC (2008). Cited2 is required for fetal lung maturation. Dev. Biol..

[28] Osada H, Tomida S, Yatabe Y, Tatematsu Y, Takeuchi T, Murakami H, Kondo Y, Sekido Y, Takahashi T (2008). Roles of achaete-scute homologue 1 in DKK1 and E-cadherin repression and neuroendocrine differentiation in lung cancer. Cancer Res..

[29] Castro DS, Martynoga B, Parras C, Ramesh V, Pacary E, Johnston C, Drechsel D, Lebel-Potter M, Garcia LG, Hunt C (2011). A novel function of the proneural factor Ascl1 in progenitor proliferation identified by genome-wide characterization of its targets. Genes Dev..

[30] Neptune ER, Podowski M, Calvi C, Cho JH, Garcia JGN, Tuder R, Linnoila RI, Tsai MJ, Dietz HC (2008). Targeted disruption of NeuroD, a proneural basic helix-loop-helix factor, impairs distal lung formation and neuroendocrine morphology in the neonatal lung. J. Biol. Chem..

[31] Borromeo MD, Savage TK, Kollipara RK, He M, Augustyn A, Osborne JK, Girard L, Minna JD, Gazdar AF, Cobb MH (2016). ASCL1 and NEUROD1 reveal heterogeneity in pulmonary neuroendocrine tumors and regulate distinct genetic programs. Cell Rep..

[32] Fujino K, Motooka Y, Hassan WA, Ali Abdalla MO, Sato Y, Kudoh S, Hasegawa K, Niimori-Kita K, Kobayashi H, Kubota I (2015). Insulinoma-associated protein 1 is a crucial regulator of neuroendocrine differentiation in lung cancer. Am. J. Pathol..

[33] Osipovich AB, Long Q, Manduchi E, Gangula R, Hipkens SB, Schneider J, Okubo T, Stoeckert CJ, Takada S, Magnuson MA (2014). Insm1 promotes endocrine cell differentiation by modulating the expression of a network of genes that includes Neurog3 and Ripply3. Development.

[34] Zhang T, Liu WD, Saunee NA, Breslin MB, Lan MS (2009). Zinc finger transcription factor INSM1 interrupts cyclin D1 and CDK4 binding and induces cell cycle arrest. J. Biol. Chem..

[35] Yu SJ, Hu JY, Kuang XY, Luo JM, Hou YF, Di GH, Wu J, Shen ZZ, Song HY, Shao ZM (2013). MicroRNA-200a promotes anoikis resistance and metastasis by targeting YAP1 in human breast cancer. Clin. Cancer Res..

[36] Kim E, Kang J, Kang M, Park J, Kim Y, Kweon T, Lee H-W, Jho E-H, Lee Y-H, Kim S-L (2020). O-GlcNAcylation on LATS2 disrupts the Hippo pathway by inhibiting its activity. Proc. Natl. Acad. Sci..

[37] Janse van Rensburg HJ, Azad T, Ling M, Hao Y, Snetsinger B, Khanal P, Minassian LM, Graham CH, Rauh MJ, Yang X (2018). The Hippo pathway component TAZ promotes immune evasion in human cancer through PD-L1. Cancer Res..

[38] Farazi TA, Brown M, Morozov P, Ten Hoeve JJ, Ben-Dov IZ, Hovestadt V, Hafner M, Renwick N, Mihailovic A, Wessels LF (2012). Bioinformatic analysis of barcoded cDNA libraries for small RNA profiling by next-generation sequencing. Methods.

[39] Hafner M, Renwick N, Farazi TA, Mihailovic A, Pena JT, Tuschl T (2012). Barcoded cDNA library preparation for small RNA profiling by next-generation sequencing. Methods.

[40] Andrews, S. FASTQC. A quality control tool for high throughput sequence data. Available online at http://www.bioinformatics.babraham.ac.uk/projects/fastqc (2010).

[41] Bolger AM, Lohse M, Usadel B (2014). Trimmomatic: A flexible trimmer for Illumina sequence data. Bioinformatics.

[42] Bray NL, Pimentel H, Melsted P, Pachter L (2016). Near-optimal probabilistic RNA-seq quantification. Nat. Biotechnol..

[43] Panarelli N, Tyryshkin K, Wong JJM, Majewski A, Yang X, Scognamiglio T, Kim MK, Bogardus K, Tuschl T, Chen YT (2019). Evaluating gastroenteropancreatic neuroendocrine tumors through microRNA sequencing. Endocr. Relat. Cancer.

[44] Livak KJ, Schmittgen TD (2001). Analysis of relative gene expression data using real-time quantitative PCR and the 2(−Delta Delta C(T)) method. Methods.

[45] Yu J, Alharbi A, Shan H, Hao Y, Snetsinger B, Rauh MJ, Yang X (2017). TAZ induces lung cancer stem cell properties and tumorigenesis by up-regulating ALDH1A1. Oncotarget.

[46] Nicol CJ, Yoon M, Ward JM, Yamashita M, Fukamachi K, Peters JM, Gonzalez FJ (2004). PPARγ influences susceptibility to DMBA-induced mammary, ovarian and skin carcinogenesis. Carcinogenesis.

[47] Raudvere U, Kolberg L, Kuzmin I, Arak T, Adler P, Peterson H, Vilo J (2019). g:Profiler: A web server for functional enrichment analysis and conversions of gene lists (2019 update). Nucleic Acids Res..

[48] Ashburner M, Ball CA, Blake JA, Botstein D, Butler H, Cherry JM, Davis AP, Dolinski K, Dwight SS, Eppig JT (2000). Gene ontology: Tool for the unification of biology. The Gene Ontology Consortium. Nat. Genet..

[49] The Gene Ontology Consortium (2019). The Gene Ontology Resource: 20 years and still GOing strong. Nucleic Acids Res..

[50] Fabregat A, Jupe S, Matthews L, Sidiropoulos K, Gillespie M, Garapati P, Haw R, Jassal B, Korninger F, May B (2018). The reactome pathway knowledgebase. Nucleic Acids Res..

[51] Shannon P, Markiel A, Ozier O, Baliga NS, Wang JT, Ramage D, Amin N, Schwikowski B, Ideker T (2003). Cytoscape: A software environment for integrated models of biomolecular interaction networks. Genome Res..

[52] Merico D, Isserlin R, Stueker O, Emili A, Bader GD (2010). Enrichment map: A network-based method for gene-set enrichment visualization and interpretation. PLoS ONE.

[53] Kucera M, Isserlin R, Arkhangorodsky A, Bader GD (2016). AutoAnnotate: A Cytoscape app for summarizing networks with semantic annotations. F1000Res.

