# Supplementary material for: A miR-375/YAP axis regulates neuroendocrine differentiation and tumorigenesis in lung carcinoid cells
Source: Sci Rep. 2021 May 17;11:10455. doi: 10.1038/s41598-021-89855-4 (PMC8129150; doi:10.1038/s41598-021-89855-4)
Supplement: Supplementary file 1 — Supplementary Information 1. [file 41598_2021_89855_MOESM1_ESM.pdf]

A miR-375/YAP Axis Regulates Neuroendocrine Differentiation and Tumorigenesis in Lung Carcinoid Cells

Xiaojing Yang[1*], Jina Nanayakkara[1*], Duncan Claypool[2], Sadegh Saghafinia[3,4], Justin JM Wong[1], Minqi Xu[1], Xiantao Wang[2], Christopher JB Nicol[5], Iacovos P Michael[3], Markus Hafner[2], Xiaolong Yang[6], and Neil Renwick[1]

[1]Laboratory of Translational RNA Biology, Department of Pathology and Molecular Medicine, 88 Stuart St, Queen's University, Kingston, ON K7L 3N6, Canada; [2]Laboratory of Muscle Stem Cells and Gene Regulation, NIAMS, 50 South Drive, Bethesda, MD 20892, USA; [3]Swiss Institute for Experimental Cancer Research, School of Life Sciences, Swiss Federal Institute of Technology Lausanne, 1015 Lausanne, Switzerland; [4]Department of Computational Biology, University of Lausanne, 1015 Lausanne, Switzerland; [5]Division of Cancer Biology and Genetics, 18 Stuart St, Queen's University, Kingston, ON K7L 3N6, Canada; [6]Cancer Research Laboratory, Department of Pathology and Molecular Medicine, 88 Stuart St, Queen's University, Kingston, ON K7L 3N6, Canada.

[*]X. Yang and J. Nanayakkara contributed equally to this work.

SUPPLEMENTARY INFORMATION

Supplementary Method. **Luciferase reporter assay.** Prior to performing the assay, miR-375 expression vector and wild-type and mutant luciferase constructs were generated. For the miR-375 expression vector, miR-375 precursor sequence was cloned into pGIPZ. To generate the wild-type luciferase construct, a putative miR-375 binding site within the YAP 3'UTR was subcloned into a pGL3-C luciferase reporter vector. To generate the mutant luciferase construct, overlapping PCR was applied to mutate the miR-375 binding site. Next, HEK293 cells were co-transfected with miR-375 expression vector or control empty vector, luciferase constructs with wild-type or mutant YAP 3'UTR, and the Renilla luciferase reporter (pRL-TK) as an internal control. Relative luciferase activity was measured using the Dual-Luciferase Reporter Assay System (Promega) and 20/20^{n} Luminometer (Turner Biosystems).

Supplementary Method. **Histologic and immunohistochemical analyses**. Formalin-fixed paraffin-embedded (FFPE) tissue blocks were generated in the Queen's Department of Pathology and Molecular Medicine. Representative tissue sections (3-4 um) were stained using the Discovery XT Automated IHC/ISH Research Platform (Ventana Medical Systems, Inc.). Histologic analyses were performed on hematoxylin and eosin (H&E)-stained tissue sections. Immunohistochemical (IHC) analyses were performed using a standard immunostaining format on a Ventana Discovery XT biomarker platform (Ventana Medical Systems, Inc.). Briefly, antigens were retrieved and detected using anti-chromogranin A, anti-synaptophysin, or anti-YAP primary antibodies, as above, in conjunction with long-chain polymer conjugated anti-mouse or anti-rabbit IgG fragments (Ventana Medical Systems, Inc.) and 3,3-diaminobenzidine color development. Specificity controls lacked primary antibodies. Slides were scanned and imaged acquired using an Olympus VS120 high resolution digital slide scanner system.

Supplementary Figure 1. Inverse miR-375/YAP expression patterns in lung carcinoid and non-NEN lung cell lines. miR-375 and YAP expression was measured by qPCR in typical and atypical lung carcinoid (H727 and H720) and non-NEN lung cell lines (NL20 and A549). **A)** Compared to non-NEN lung cells, miR-375 was higher expressed in lung carcinoid cells. **B)** YAP demonstrated inverse expression patterns compared to miR-375.

Supplementary Figure 2. Quantification of neuroendocrine marker expression in miR-375 depleted H727 cells. CgA and SYP expression was significantly lower in miR-375 depleted H727 cells (n=4) compared to empty vector control cells (n=4) (t-test: *P<0.05). Densitometry analysis was applied to the Western blot from Figure 3A.

Supplementary Figure 3. Neuroendocrine marker expression in miR-375 depleted xenograft tumors. CgA and SYP expression was lower in miR-375 depleted xenografts compared to controls. No antibody control presented for reference; scale bar represents 100 μm.

Supplementary Figure 4. Luciferase reporter assay for miR-375/YAP binding. Co-transfection of miR-375 expression vector and wild-type but not mutant YAP 3'UTR construct reduced luciferase reporter activity in HEK293. n=4 to 7 biological replicates for each condition.

Supplementary Figure 5. Quantification of neuroendocrine marker expression in YAP overexpression H727 cells. CgA and SYP expression was significantly lower in H727-YAP-S127A (+) Dox cells (n=4) compared to (-) Dox cells (n=4) (t-test: *P<0.05, **P<0.01). Densitometry analysis was applied to the Western blot from Figure 5A.

Supplementary Figure 6. Quantification of neuroendocrine marker expression in YAP overexpression xenograft tumors. CgA and SYP expression was significantly lower in H727-YAP-S127A (+) Dox xenografts (n=3) compared to (-) Dox xenografts (n=3) (t-test: *P<0.05). Densitometry analysis was applied to the Western blot from Figure 5H.

Supplementary Figure 7. Neuroendocrine marker expression in YAP overexpression xenograft tumors. Immunohistochemical staining of CgA and SYP showed lower expression in H727-YAP-S127A (+) Dox xenografts compared to (-) Dox xenografts. No antibody control presented for reference; scale bar represents 100 μm.

Supplementary Figure 8. Gene expression of neuroendocrine transcription factors after siYAP knockdown in miR-375 depleted cells. Real-time PCR (n=3) of neuroendocrine transcription factors (ASCL1, INSM1, NEUROD1) demonstrated partial recovery of gene expression after siYAP knockdown in miR-375 depleted H727 cells. The variation in gene expression after siYAP may be explained by miR-375 independent transcriptional regulation through YAP. Expression is relative to empty vector control H727 cells.

Supplementary Figure 9. miR-375/YAP gene signature in human lung carcinoid tissue. Gene expression data for a well differentiated molecular subtype (LC1) of lung carcinoid was extracted from a recent study of human lung carcinoid tissue by Laddha et al.[22] (n=15). Genes were ranked by increasing expression level and plotted against expression value ($\log_2$ TPM). While YAP expression was low, neuroendocrine transcription factors (ASCL1, INSM1) were inversely highly expressed except for NEUROD1. Epithelial-related genes and tumor suppressors (GPRC5A, ANXA1, CITED2) were also lowly expressed.

Supplementary Table 1. gRNA oligonucleotide sequences for CRISPR/Cas9 editing. miR-375-gRNA-1 and miR-375-gRNA-2 were designed to direct CRISPR/Cas9 cleavage of the Drosha and Dicer processing sites in the miR-375 precursor sequence.

Supplementary Table 2. Sanger sequencing primers for amplifying miR-375 gene. Sanger sequencing primers were designed to confirm miR-375 gene editing.

Supplementary Table 3. Real-time PCR primers for genes regulated by miR-375/YAP. Primers were designed for real-time PCR validation of genes regulated by the miR-375/YAP axis.

Supplementary Table 4. Pathway enrichment of dysregulated genes following miR-375 depletion. After miR-375 depletion, 788 genes were upregulated and 751 genes were downregulated. Upregulated genes were enriched in 51 pathways, mostly related to secretion and exocytosis; downregulated genes were enriched in 182 pathways mostly related to Notch signalling and cell cycle. To define dysregulated genes, a 1.5-fold threshold for changes in gene expression was applied. Abbreviation: undefined (und).

Supplementary Table 5. Ranked miR-375 target prediction. Using the Bio-miRTa target prediction algorithm, predicted miR-375 targets (n=2,677) from six widely used miRNA targeting databases were pooled and assigned a predicted targeting score (PS) as described [7]. Next, a biological targeting score (BS) was assigned based on gene expression fold-change of predicted targets after miR-375 depletion. Subsequently, a final targeting score (FS) was calculated by combining predicted and biological targeted scores. Top-ranked targets included PLEKHA3, YAP, and BCL1.

Supplementary Table 6. Pathway enrichment of dysregulated genes following YAP overexpression. After YAP overexpression, 937 genes were upregulated and 1032 genes were downregulated. Upregulated genes were enriched in 237 pathways, mostly related to exocytosis and cytoskeletal organization; downregulated genes were enriched in 26 pathways mostly related to hormone secretion. To define dysregulated genes, a 1.5-fold threshold for changes in gene expression was applied.

Supplementary Table 7. Pathway enrichment of dysregulated genes following miR-375 depletion and YAP overexpression. After intersecting dysregulated genes from miR-375 depletion and YAP overexpression experiments, 218 genes were upregulated and 217 genes were downregulated. Upregulated genes were enriched in 46 pathways, mostly related to cell motility, epithelial development and exocytosis; downregulated genes were enriched in 18 pathways mostly related to neural differentiation and hormone secretion.

Fig. S1

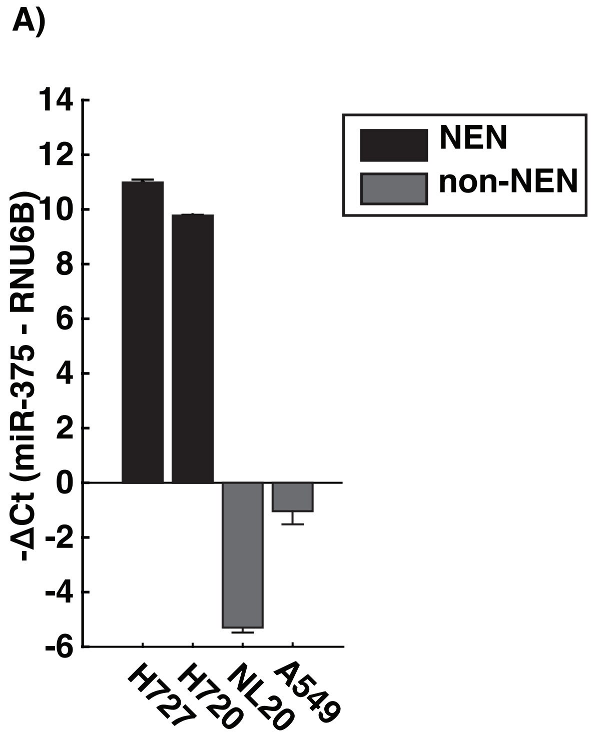
A)
-ΔCt (miR-375 - RNU6B)
14
12
10
8
6
4
2
0
-2
-4
-6
NEN
non-NEN
H727
H720
NL20
A549

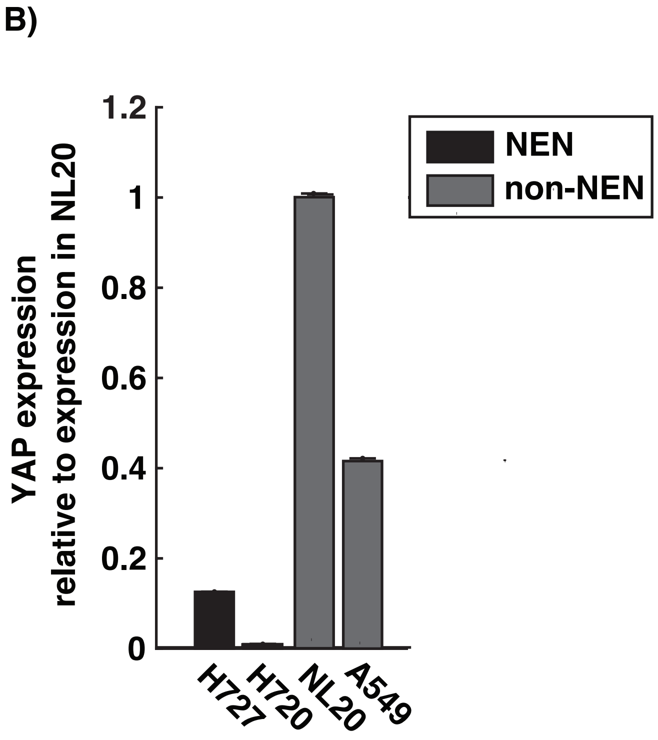
B)
YAP expression
relative to expression in NL20
1.2
1
0.8
0.6
0.4
0.2
0
NEN
non-NEN
H727
H720
NL20
A549

Fig. S2

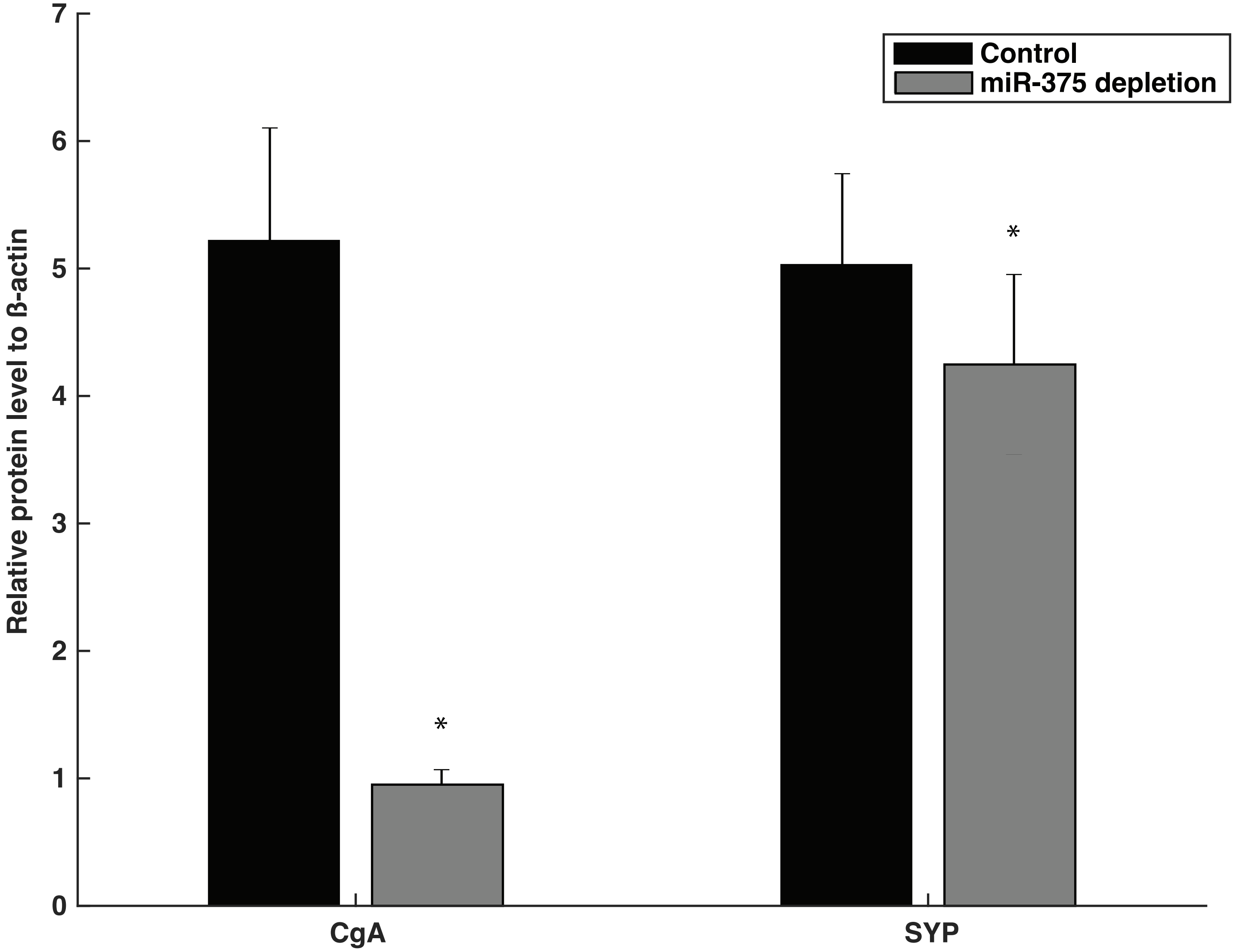

Control
miR-375 depletion
Relative protein level to β-actin
0
1
2
3
4
5
6
7
*
*
CgA
SYP

Fig. S3

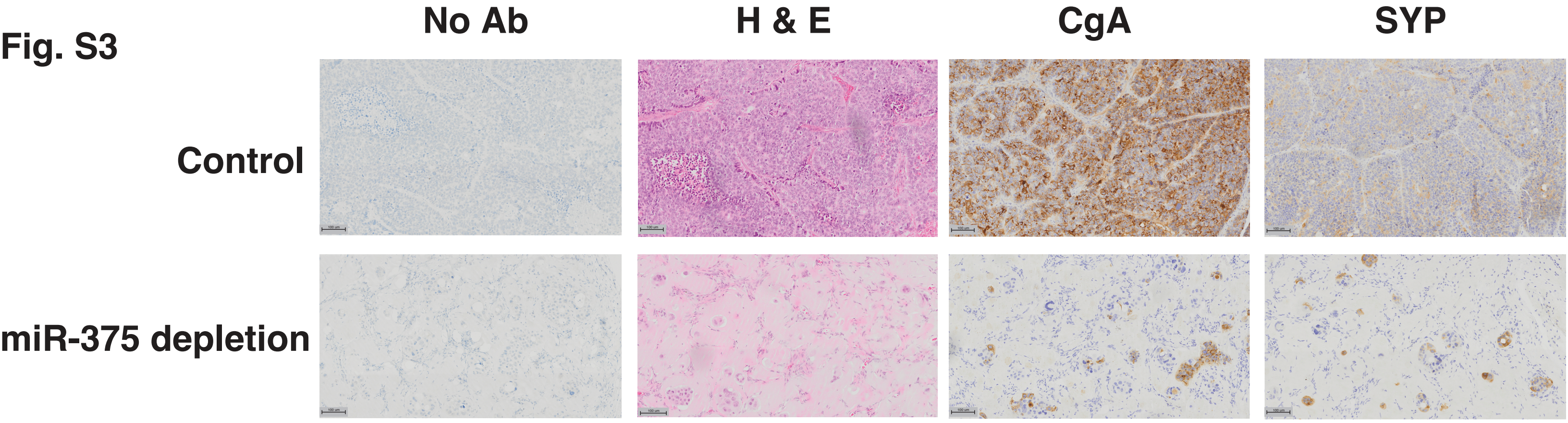

Fig. S4

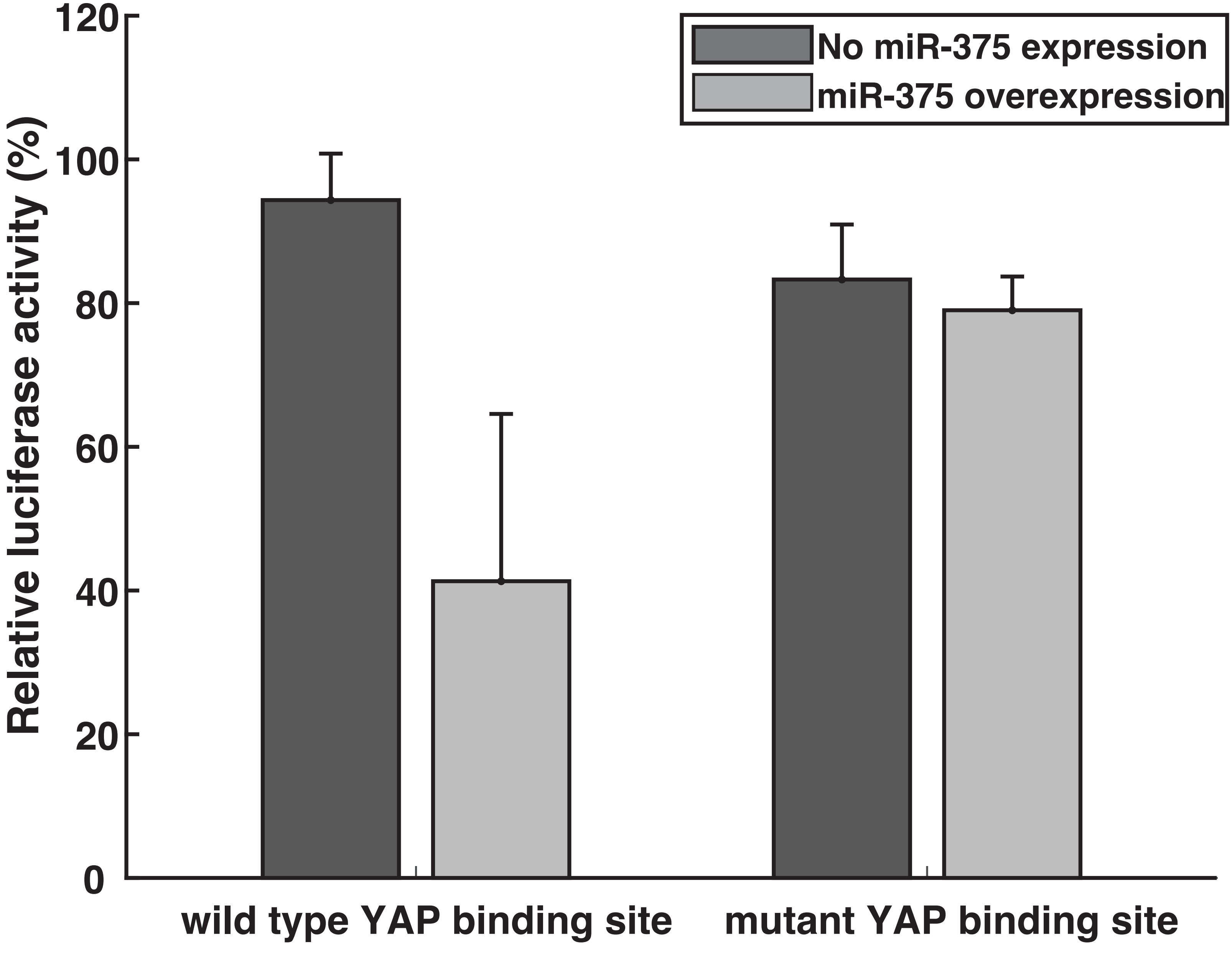

120
100
80
60
40
20
0
Relative luciferase activity (%)
No miR-375 expression
miR-375 overexpression
wild type YAP binding site
mutant YAP binding site

Fig. S5

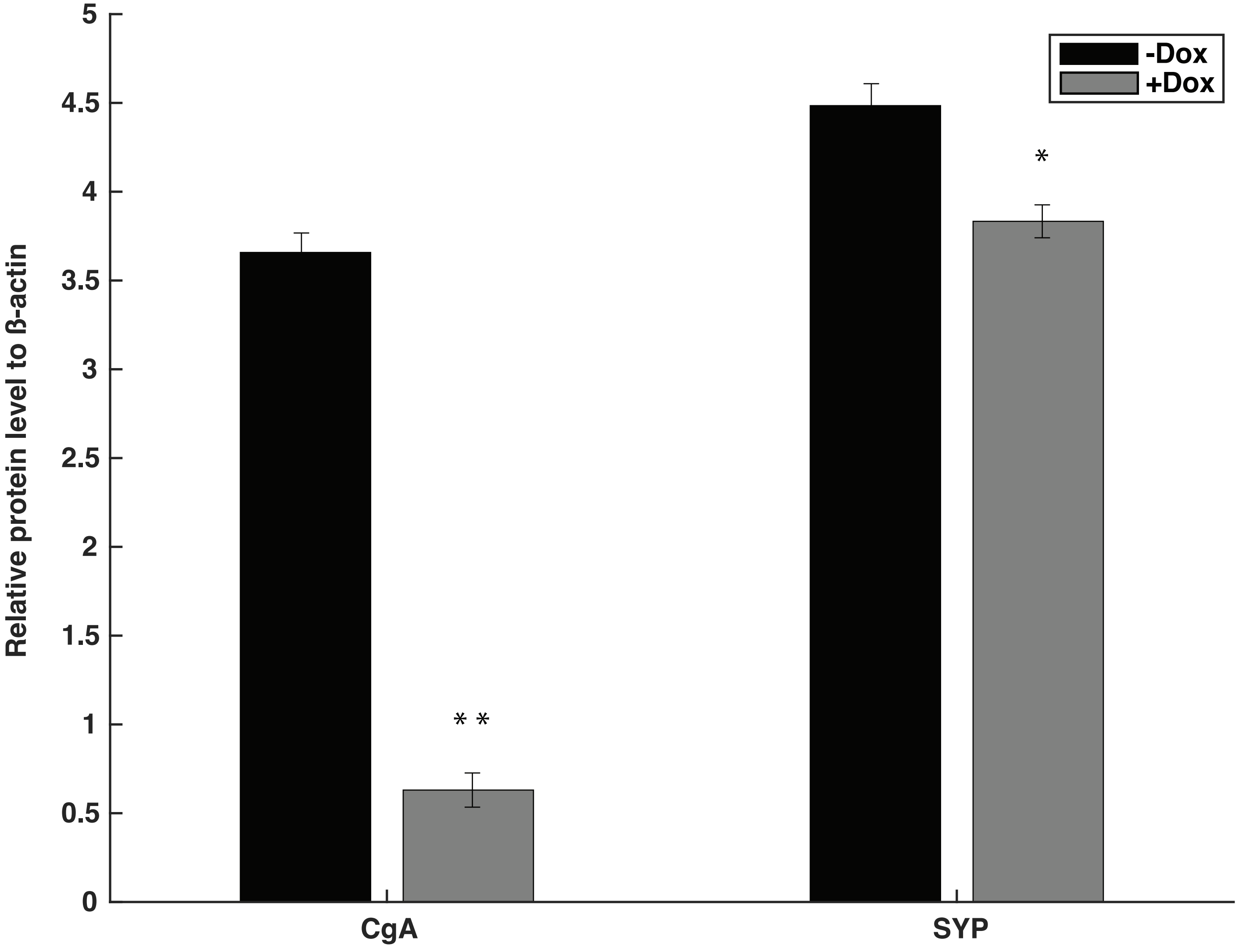
Relative protein level to β-actin
5
4.5
4
3.5
3
2.5
2
1.5
1
0.5
0
-Dox
+Dox
* *
*
CgA
SYP

Fig. S6

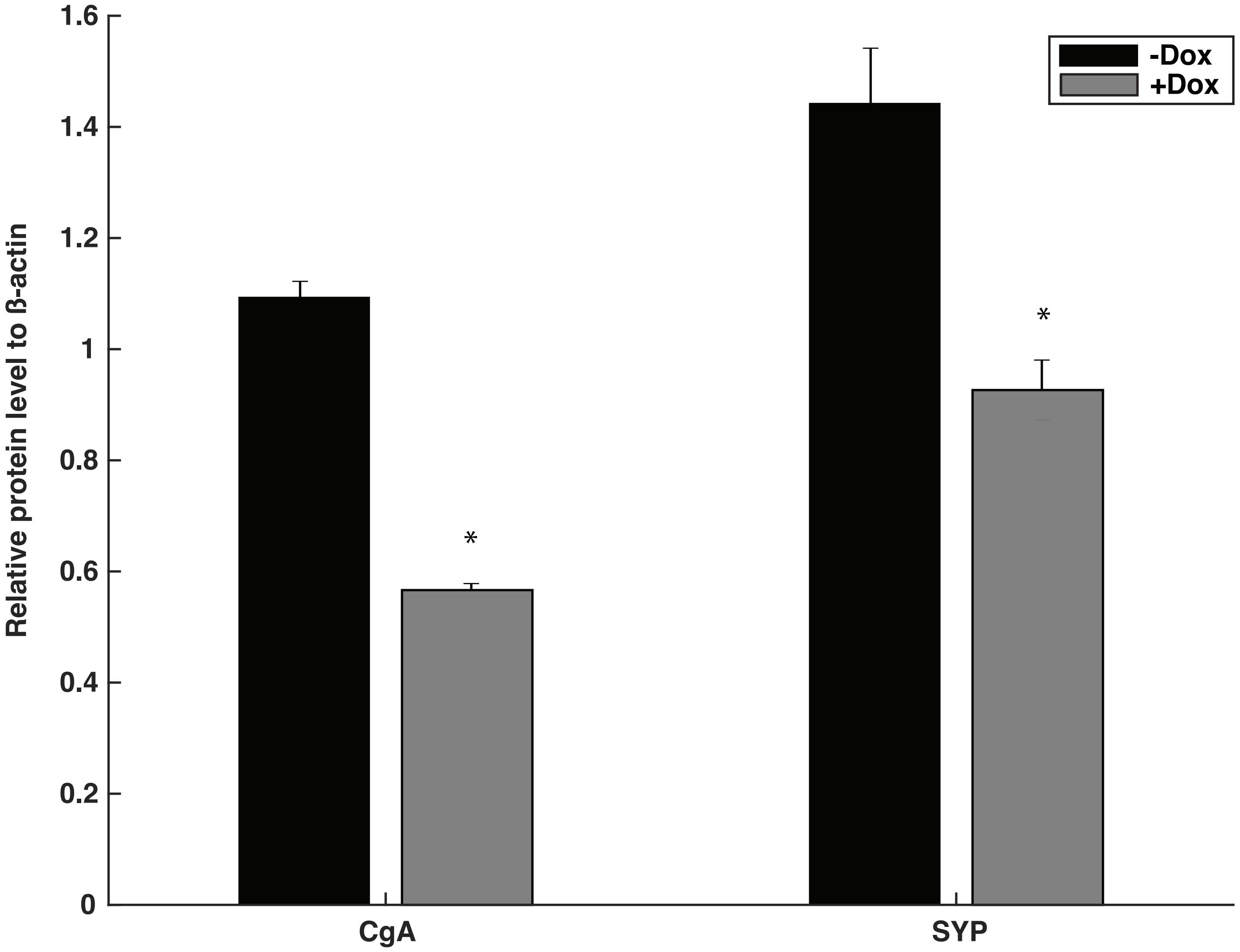
Relative protein level to ß-actin
1.6
1.4
1.2
1
0.8
0.6
0.4
0.2
0
-Dox
+Dox
*
*
CgA
SYP
