# Supplementary material for: A miR-375/YAP axis regulates neuroendocrine differentiation and tumorigenesis in lung carcinoid cells
Source: Sci Rep. 2021 May 17;11:10455. doi: 10.1038/s41598-021-89855-4 (PMC8129150; doi:10.1038/s41598-021-89855-4)
Supplement: Supplementary file 2 — Supplementary Information 2. [file 41598_2021_89855_MOESM2_ESM.pdf]

Fig. S7

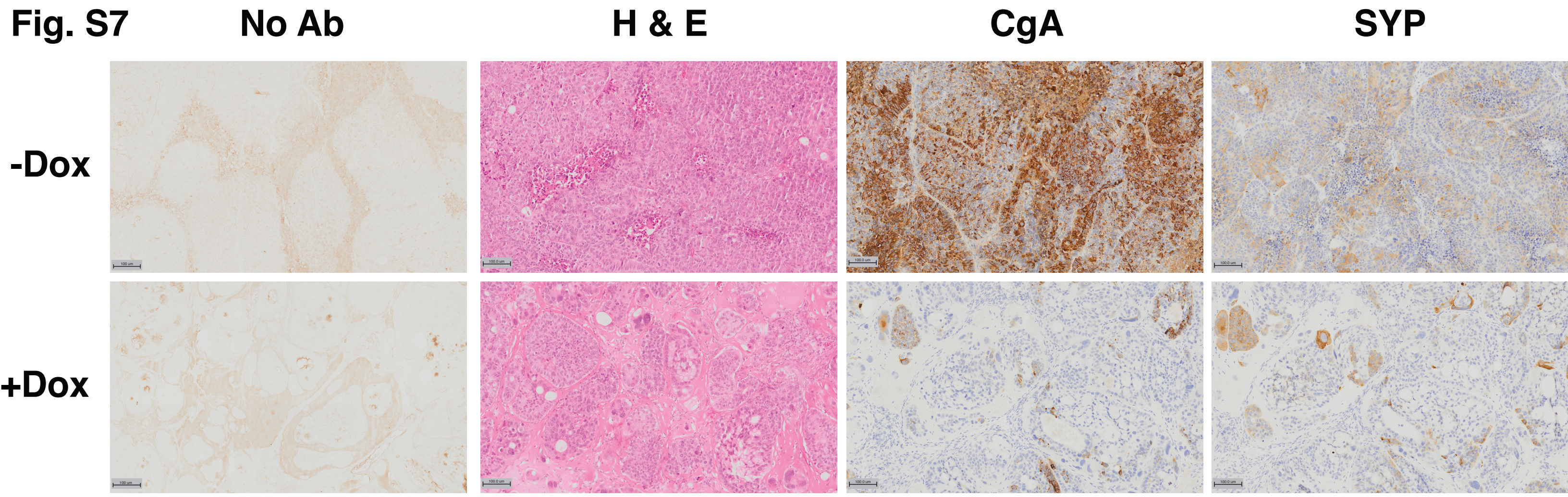

Fig. S8

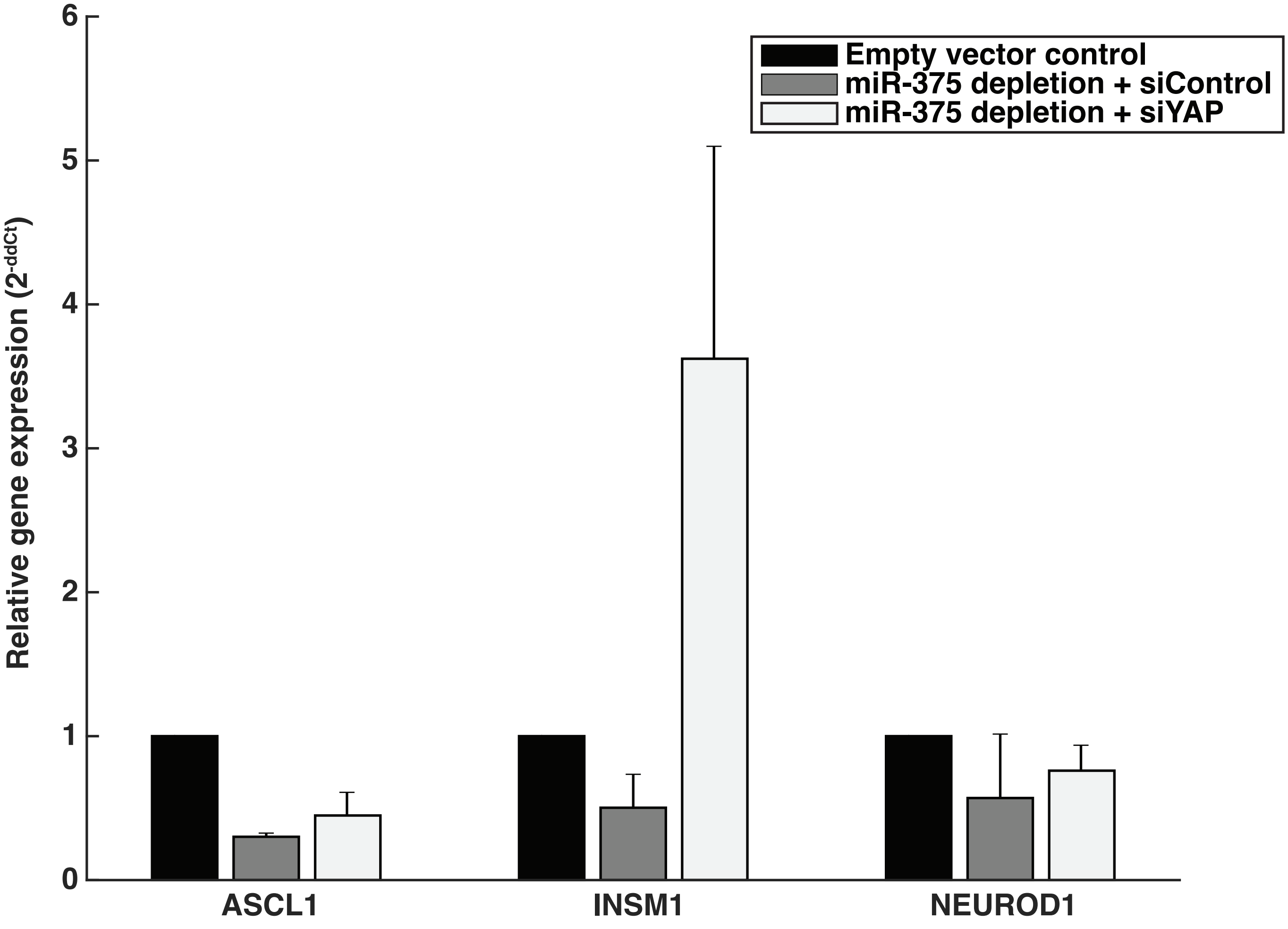
Empty vector control
miR-375 depletion + siControl
miR-375 depletion + siYAP
Relative gene expression (2-ddCt)
0
1
2
3
4
5
6
ASCL1
INSM1
NEUROD1

Fig. S9

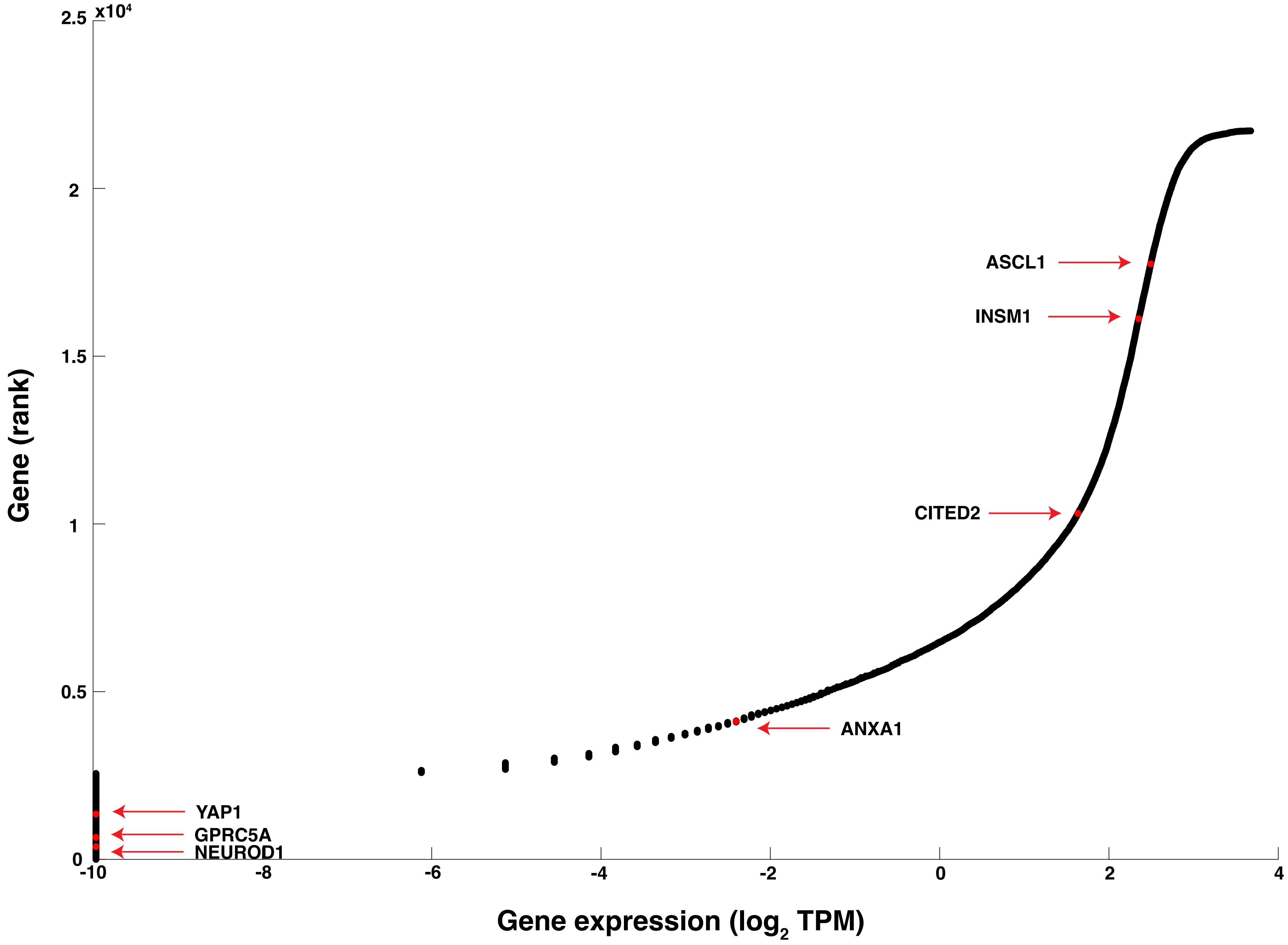
x10^4
2.5
2
1.5
1
0.5
0
Gene (rank)
ASCL1
INSM1
CITED2
ANXA1
YAP1
GPRC5A
NEUROD1
-10
-8
-6
-4
-2
0
2
4
Gene expression (log2 TPM)

**Supplementary Table 1. sgRNA oligonucleotide sequences for CRISPR/Cas9 editing.**

| sgRNA Oligos | Forward Primer (5′–3′) | Reverse Primer (5′–3′) |
|---|---|---|
| miR-375-sgRNA-1 | GACGAGCCCCTCGCACAAAC | GTTTGTGCGAGGGGCTCGTC |
| miR-375-sgRNA-2 | TTGTTCGTTCGGCTCGCGTG | CACGCGAGCCGAACGAACAA |

**Supplementary Table 2: Sanger sequencing primers for amplifying miR-375 gene**

| | Primer sequence |
|---|---|
| Forward Primer (5′–3′) | GAGATCCGAGGAGTGGATTTG |
| Reverse Primer (5′–3′) | GGGACTGAACAGGCAGTATAAG |

**Supplementary Table 3: Real-time PCR primers for genes regulated by miR-375/YAP**

| Gene | Forward Primer (5′–3′) | Reverse Primer (5′–3′) |
|---|---|---|
| GPRC5A | GCTGCTCACAAAGCAACGAA | ATAGAGCGTGTCCCCTGTCT |
| ANXA1 | TGCAAGAAGGTAGAGATAAAGACAC | GCGACATCCGAGGATGGATT |
| CITED2 | AAAGGGAACGGCTCCGAATC | TGCCATTTCCAGTCCTTCCG |
| ASCL1 | CGCTCGGCGGTCGAGTA | GTTGTGCGATCACCCTGCTT |
| INSM1 | TACGCGTTTGTCTCGTGGTT | CAGAGATTGGTAGGCGAGGC |
| NEUROD1 | ATAGACCTGCTAGCCCCTCA | TGGTCATGTTTCGATTTCCTTTGTT |
