# Supplementary material for: A miR-375/YAP axis regulates neuroendocrine differentiation and tumorigenesis in lung carcinoid cells
Source: Sci Rep. 2021 May 17;11:10455. doi: 10.1038/s41598-021-89855-4 (PMC8129150; doi:10.1038/s41598-021-89855-4)
Supplement: Supplementary file 3 — Supplementary Information 3. [file 41598_2021_89855_MOESM3_ESM.pdf]

FULL LENGTH GEL AND BLOT IMAGES

All replicates for Western blots are presented below. Representative sections of Western blots are highlighted in dashed red rectangle borders. These sections are presented in the figures within the main text. If relevant, images of the same Western blot with different image exposure times are presented side-by-side.

Figure 1B

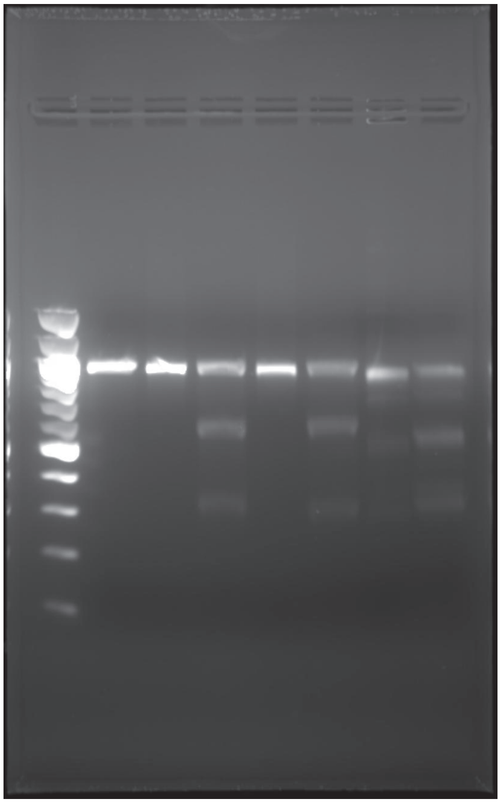

Figure 3A

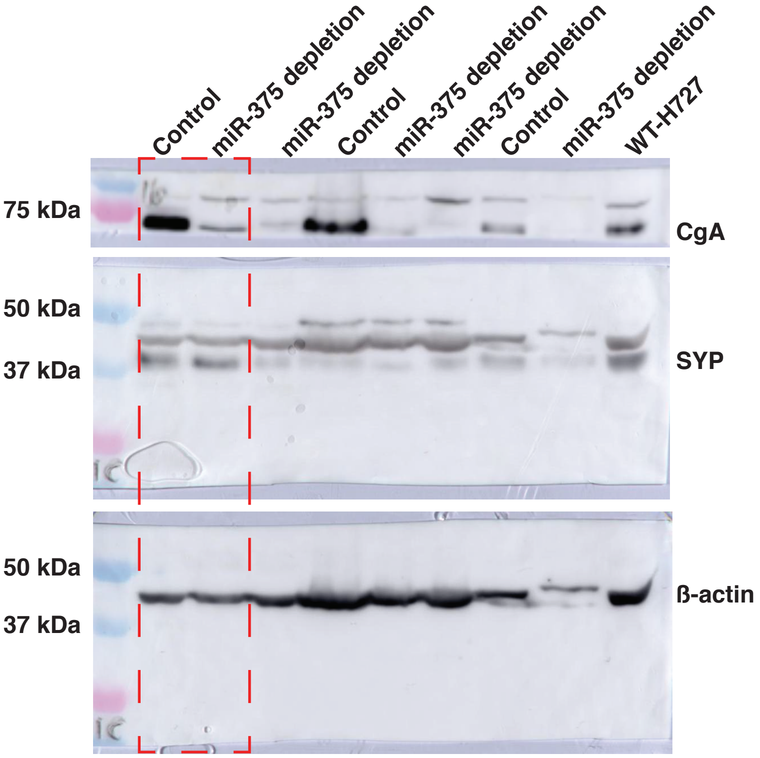

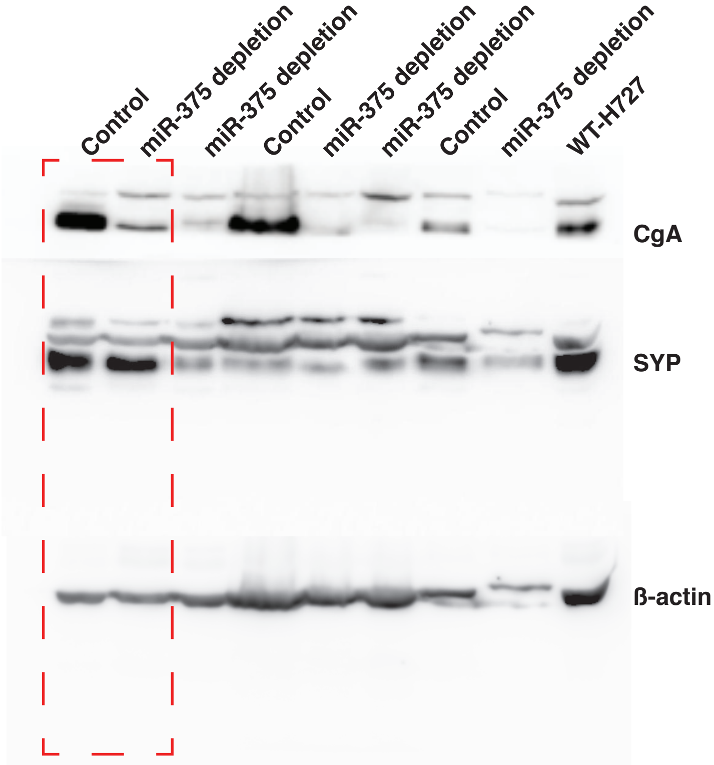

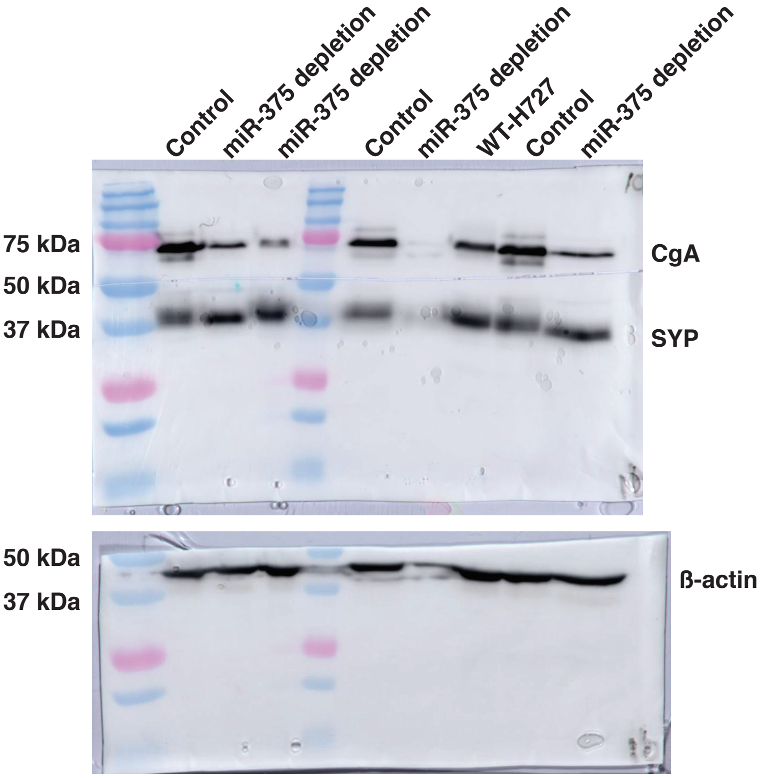
Control
miR-375 depletion
miR-375 depletion
Control
miR-375 depletion
WT-H727
Control
miR-375 depletion
75 kDa
50 kDa
37 kDa
CgA
SYP
50 kDa
37 kDa
ß-actin

Figure 5A

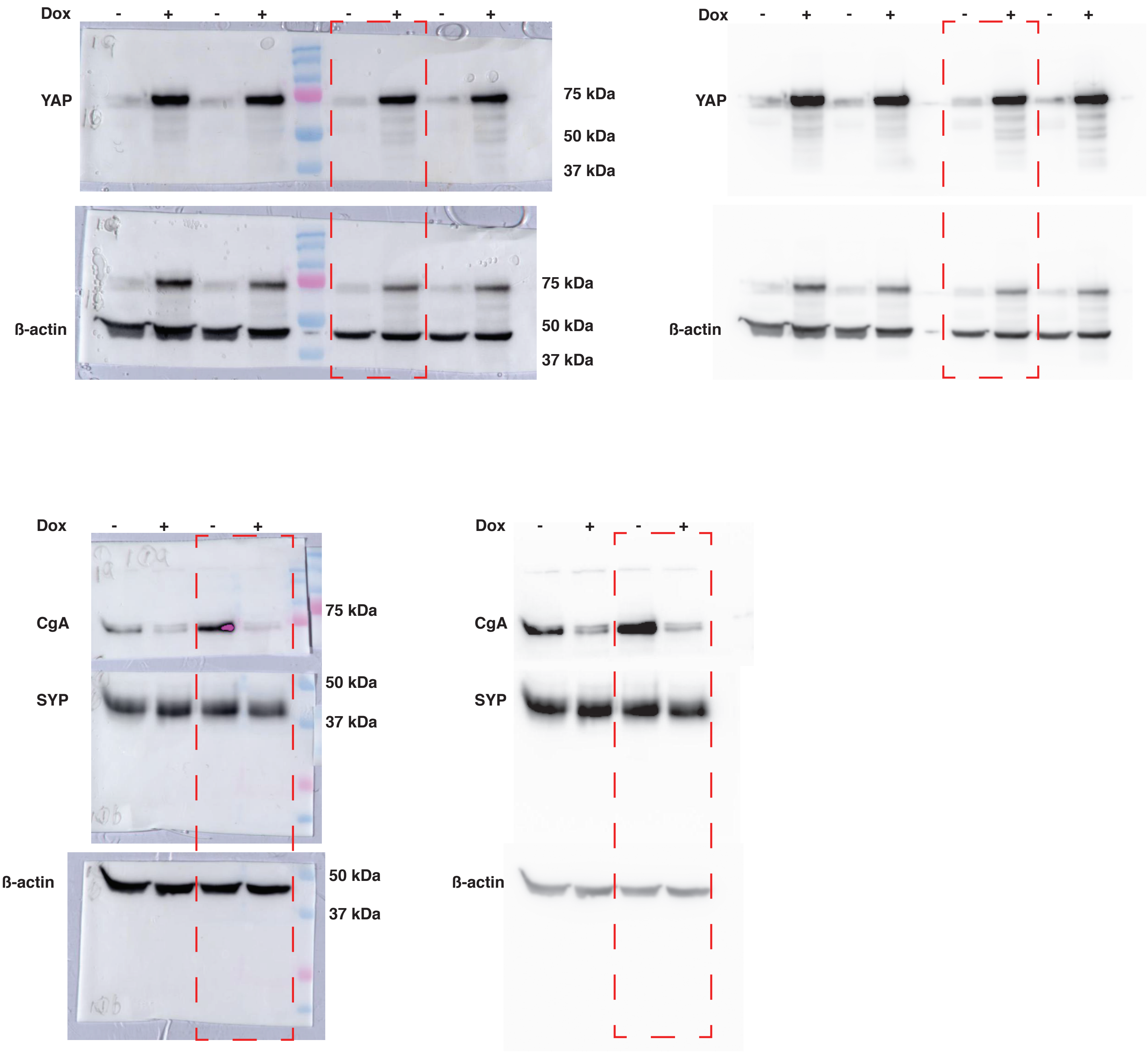

Dox
-
+
-
+
-
+
-
+
YAP
75 kDa
50 kDa
37 kDa
ß-actin
75 kDa
50 kDa
37 kDa
Dox
-
+
-
+
-
+
-
+
YAP
ß-actin
Dox
-
+
-
+
CgA
75 kDa
SYP
50 kDa
37 kDa
ß-actin
50 kDa
37 kDa
Dox
-
+
-
+
CgA
SYP
ß-actin

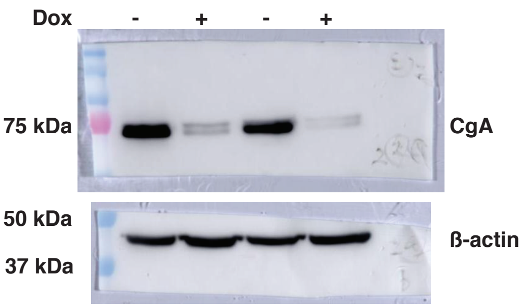
Dox
-
+
-
+
75 kDa
CgA
50 kDa
37 kDa
ß-actin

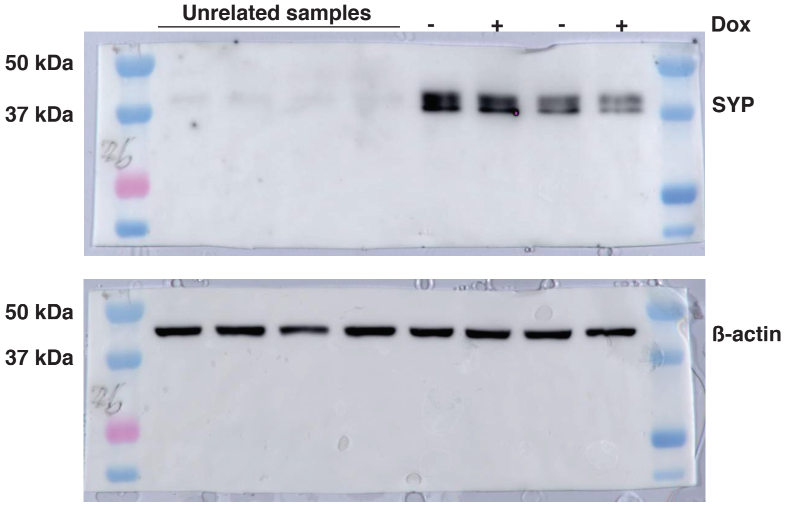
Unrelated samples
-
+
-
+
Dox
50 kDa
37 kDa
SYP
50 kDa
37 kDa
ß-actin

Figure 5H

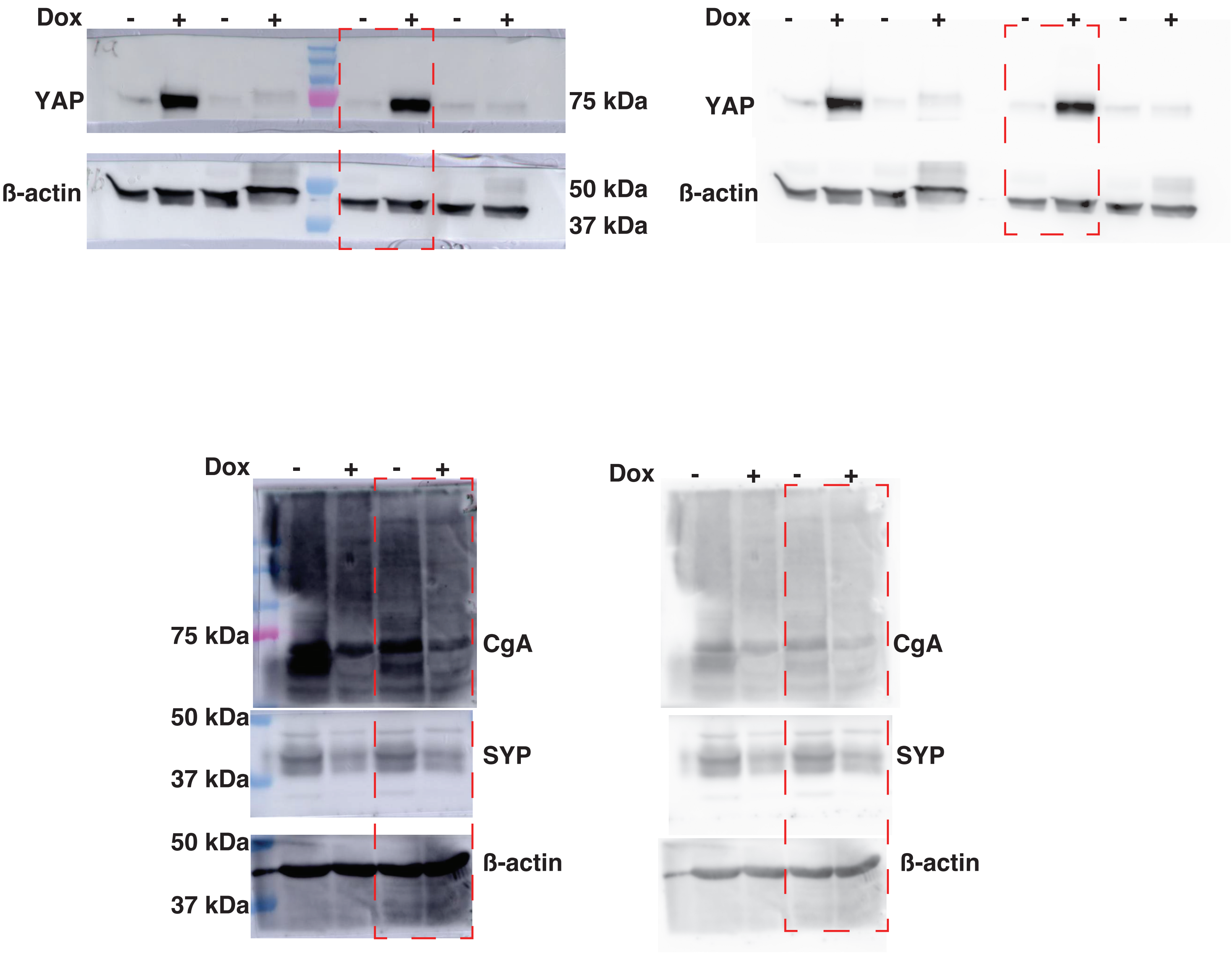

Dox - + - + - + - +
YAP
ß-actin
75 kDa
50 kDa
37 kDa
Dox - + - + - + - +
YAP
ß-actin
Dox - + - +
75 kDa
50 kDa
37 kDa
50 kDa
37 kDa
CgA
SYP
ß-actin
Dox - + - +
CgA
SYP
ß-actin

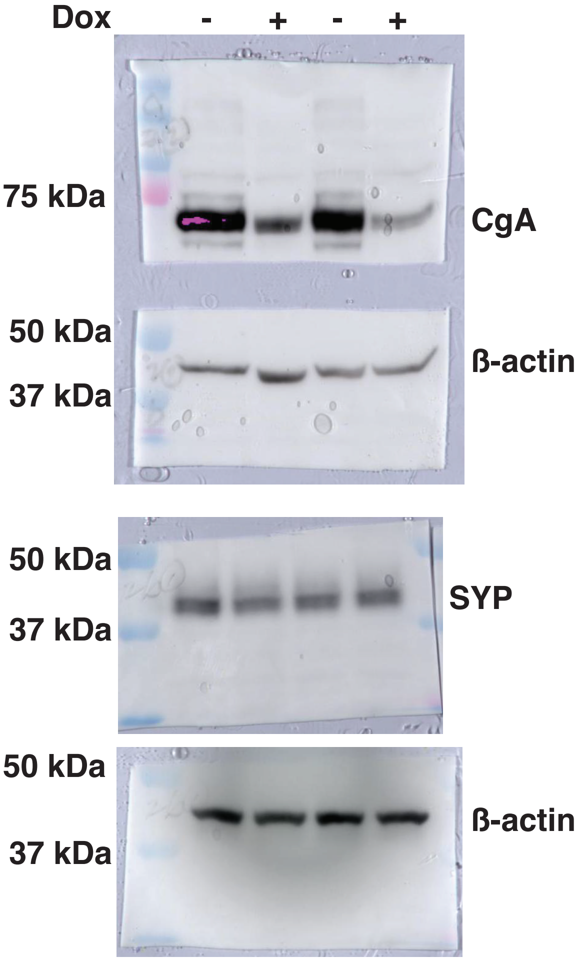
Dox
-
+
-
+
75 kDa
CgA
50 kDa
ß-actin
37 kDa
50 kDa
SYP
37 kDa
50 kDa
ß-actin
37 kDa

Figure 6A

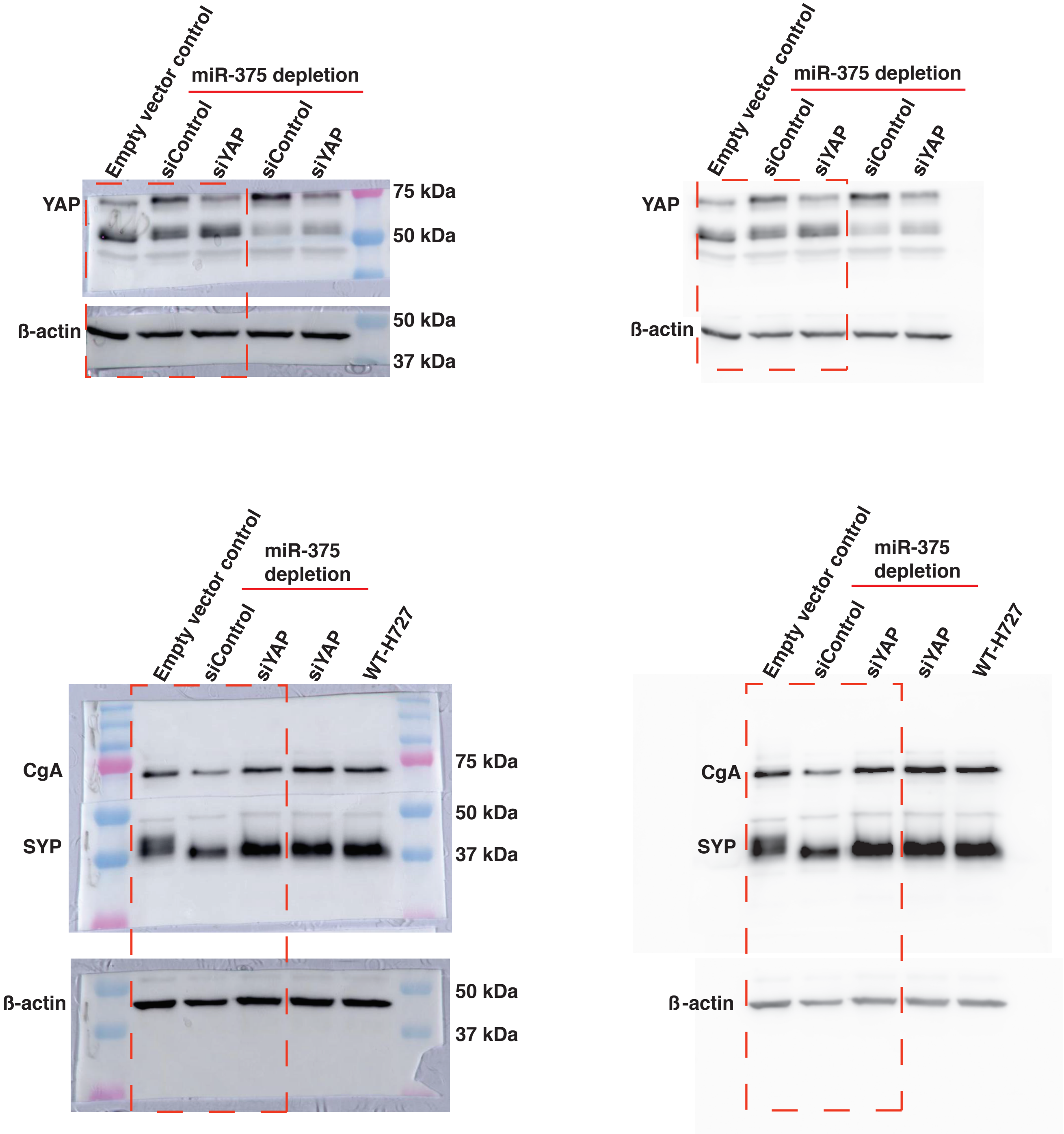

Empty vector control
miR-375 depletion
siControl
siYAP
siControl
siYAP
YAP
75 kDa
50 kDa
ß-actin
50 kDa
37 kDa
Empty vector control
miR-375 depletion
siControl
siYAP
siControl
siYAP
YAP
ß-actin
Empty vector control
miR-375 depletion
siControl
siYAP
siYAP
WT-H727
CgA
SYP
75 kDa
50 kDa
37 kDa
ß-actin
50 kDa
37 kDa
Empty vector control
miR-375 depletion
siControl
siYAP
siYAP
WT-H727
CgA
SYP
ß-actin

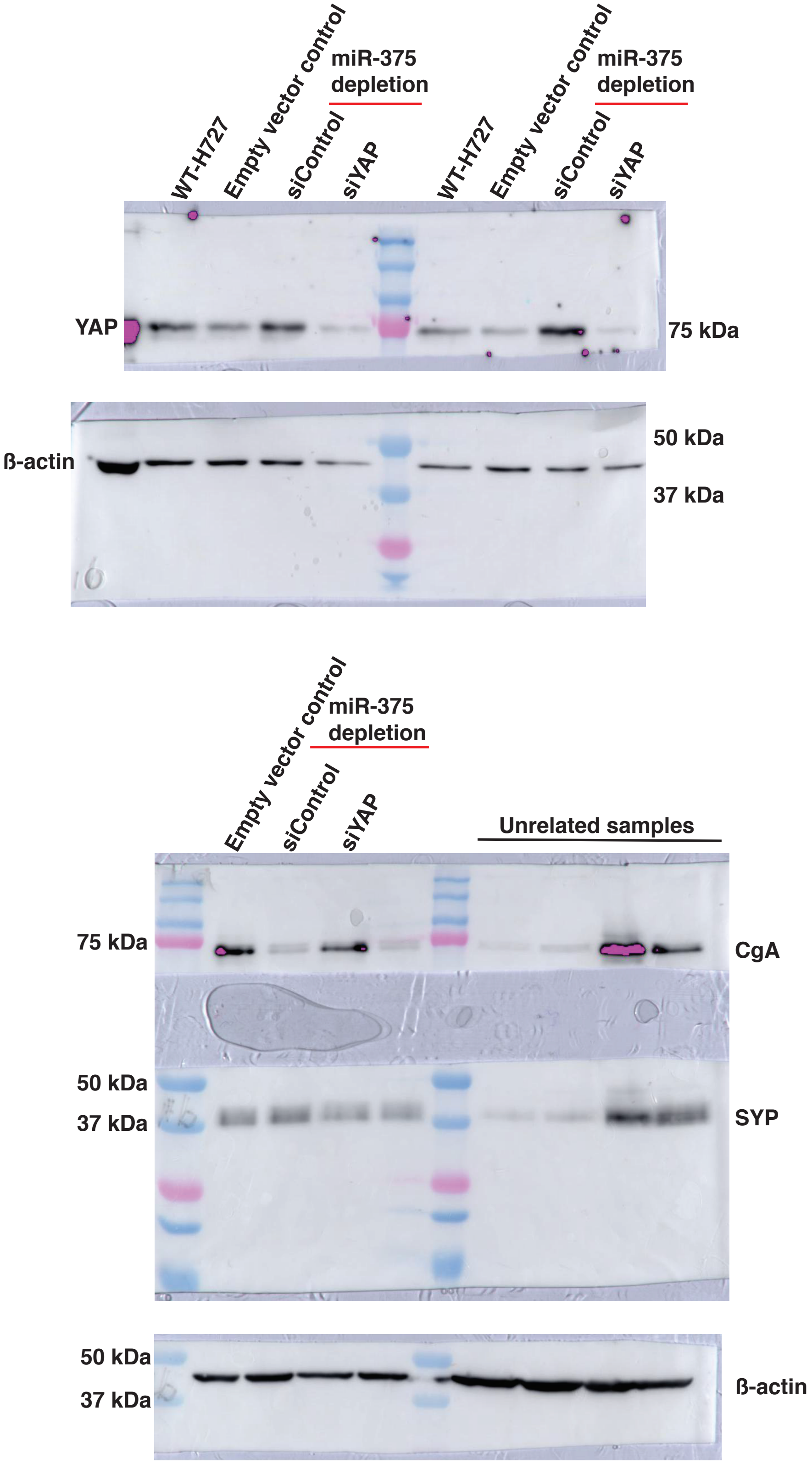
WT-H727
Empty vector control
siControl
siYAP
miR-375
depletion
WT-H727
Empty vector control
siControl
siYAP
miR-375
depletion
YAP
75 kDa
ß-actin
50 kDa
37 kDa
Empty vector control
siControl
siYAP
miR-375
depletion
Unrelated samples
75 kDa
CgA
50 kDa
37 kDa
SYP
50 kDa
37 kDa
ß-actin
